# Exceptional electrocatalytic oxygen evolution via tunable charge transfer interactions in La_0.5_Sr_1.5_Ni_1−*x*_Fe_*x*_O_4±*δ*_ Ruddlesden-Popper oxides

**DOI:** 10.1038/s41467-018-05600-y

**Published:** 2018-08-08

**Authors:** Robin P. Forslund, William G. Hardin, Xi Rong, Artem M. Abakumov, Dmitry Filimonov, Caleb T. Alexander, J. Tyler Mefford, Hrishikesh Iyer, Alexie M. Kolpak, Keith P. Johnston, Keith J. Stevenson

**Affiliations:** 1 Department of Chemistry, The University of Texas at Austin, 1 University Station, Austin, TX 78712 USA; 2 Texas Materials Institute, The University of Texas at Austin, 1 University Station, Austin, TX 78712 USA; 3 Department of Mechanical Engineering, Massachusetts Institute of Technology, Cambridge, MA 02139 USA; 4 Center for Electrochemical Energy Storage, Skolkovo Institute of Science and Technology, 3 Nobel Street, Moscow, 143026 Russia; 5 Department of Chemistry, Moscow State University, 1 Leninskiye Gory, Moscow, 119991 Russia; 6 McKetta Department of Chemical Engineering, The University of Texas at Austin, 1 University Station, Austin, TX 78712 USA; 7 Present Address: Exponent Failure Analysis Associates, 9 Strathmore Rd, Natick, MA 01760 USA; 8 Present Address: Department of Materials Science & Engineering, Stanford University, 496 Lomita Mall, Stanford, CA 94305 USA

## Abstract

The electrolysis of water is of global importance to store renewable energy and the methodical design of next-generation oxygen evolution catalysts requires a greater understanding of the structural and electronic contributions that give rise to increased activities. Herein, we report a series of Ruddlesden–Popper La_0.5_Sr_1.5_Ni_1−*x*_Fe_*x*_O_4±*δ*_ oxides that promote charge transfer via cross-gap hybridization to enhance electrocatalytic water splitting. Using selective substitution of lanthanum with strontium and nickel with iron to tune the extent to which transition metal and oxygen valence bands hybridize, we demonstrate remarkable catalytic activity of 10 mA cm^−2^ at a 360 mV overpotential and mass activity of 1930 mA mg^−1^_ox_ at 1.63 V via a mechanism that utilizes lattice oxygen. This work demonstrates that Ruddlesden–Popper materials can be utilized as active catalysts for oxygen evolution through rational design of structural and electronic configurations that are unattainable in many other crystalline metal oxide phases.

## Abstract (web summary)

Water electrolysis provides a potential means to large-scale renewable fuel generation, although sluggish oxygen evolution kinetics challenges progress. Here, authors report on Ruddlesden–Popper oxides as active oxygen evolution electrocatalysts that provide impetus for overcoming kinetic barriers.

## Introduction

Increasing global energy demand requires greater efficiency in water electrolyzers to generate hydrogen at a low cost as well as in other devices for energy storage such as rechargeable metal-air batteries. Given that the efficiencies of devices for these types of energy storage are limited largely by the slow kinetics of the oxygen evolution reaction (OER, 4OH^–^ → O_2_ + 2H_2_O + 4e^–^), extensive work has been done to reduce the overpotential required to evolve oxygen in basic electrolytes using state-of-the-art catalysts^1^. While precious metals such as Ir and Ru have become standard OER catalysts when working at low pH, alkaline electrolytes allow for the use of abundant, less expensive metals. For example, Ni–M oxyhydroxides such as Ni_1−*x*_Fe_*x*_OOH are known to be very active for the OER; however, lack of long-range order and uncontrolled electronic structure stemming from different synthetic methods^2^ complicates elucidation of the mechanism(s) by which the OER activity is improved^3–5^. In fact, recent reports question whether Fe is part of the catalytic cycle or if it promotes partial charge transfer between Ni and Fe metal centers^2,4,6^. Additionally, recent work on Ni–Fe oxyhydroxides demonstrated that a significant portion of the measured OER current may be due to other processes and highlighted the need for careful electrochemical analysis of what reactions are contributing to the high activities reported^7^. Collectively this means that while Ni_1−*x*_Fe_*x*_OOH materials have been reported as highly active catalysts, the large variations in electronic configuration and the resulting catalytic activity in these studies complicate establishment of precise structure–property correlations for Ni–Fe oxyhydroxides^2,5,8^.

In contrast, perovskite oxides with the nominal formula ABO_3±*δ*_, in which A is a rare-earth or alkaline earth element and B is a transition metal, have recently been shown to promote OER catalysis through increased ionic and electronic conductivities, as well as good structural stability and synthetic versatility, all of which enable the development of rational catalyst design criteria^9–11^. Importantly, the ability to substitute elements of varying valence, electronegativity, or ionic size into A- and B-site directly influences the resultant properties of these catalysts^1,12^. An example of this was the substitution of Cu into the A-site of the quadruple perovskite CaCu_3_Fe_4_O_12_ to increase stability of the catalyst^13^. Recently, we demonstrated that highly covalent Co 3*d*–O 2*p* bonding in SrCoO_2.7_ improved OER activity via a more energetically favorable lattice oxygen mediated (LOM) reaction pathway, consistent with prior reports and theory^14–16^. This LOM mechanism does not require the redox switching of transition metal sites, but rather utilizes lattice oxygen in the OER when the Fermi level $(E_{\mathrm F}^0)$ crosses the transition metal 3*d*–O 2*p* hybridized bands. This results in ligand holes that activate lattice oxygen which may combine with chemisorbed OH to produce O_2_−. This mechanism has since been confirmed by others using isotopic labeling experiments to detect exchanged oxygen from the lattice of SrCoO_3_^17^. In a separate report, SrNiO_3_ perovskite was predicted to be more covalent, even more prone to oxygen deficiency, and thus be more catalytically active than SrCoO_3_^18^. Unfortunately, this prediction is hard to validate experimentally as SrNiO_3_ adopts a non-perovskite structure based on a hexagonal close packing of Sr and O atoms^19^. In addition, it is known that the substitution of Sr into LaNiO_3_ is limited to ~5–20% after which phase impurities appear that result in poor catalytic activity^20,21^.

To overcome this limitation, we investigate an alternative crystal structure to the perovskite-phase SrNiO_3_ that promotes high OER activity but does not suffer from limited Sr solubility and can stabilize highly oxidized, covalently bound Ni. The Ruddlesden–Popper (RP) crystal structure is represented as A_*n*+1_B_*n*_O_3*n*+1_ or equivalently (AO)(ABO_3±δ_)_*n*_, wherein perovskite layers with a thickness of *n* (BO_6_) octahedra are separated by rocksalt (AO)(OA) double layers. This RP phase can accommodate all the elemental substitutions available to perovskites as well as additional compositions that are not stable in the perovskite structure^22^. While Ruddlesden–Popper materials have been explored as solid oxide fuel-cell cathodes owing to their chemical flexibility and labile lattice oxygen, they have not been fully examined for room temperature water oxidation^23–29^.

Herein, we report a series of La_0.5_Sr_1.5_Ni_1−*x*_Fe_*x*_O_4±*δ*_ (LSNF, *x* = 0 to 1) OER catalysts that have enabled us to achieve exceptionally high catalytic activities at low overpotentials with small OER Tafel slopes. We show that Sr substitution promotes high catalytic activity by further oxidizing Ni via charge compensation, enhancing Ni–O covalency and electronic conductivity. Chemical substitution of Fe for Ni introduces and tunes the overlap between the Ni and Fe 3*d* bands and the O *2p* band. Density functional theory (DFT) modeling confirms that cross-gap hybridization between *e*_*g*_(Ni), *p*(O) and *e*_*g*_(Fe) bands across the Fermi level enhances charge transfer interactions across Fe–O–Ni bridges and the bandwidth available for electrode-adsorbate electron transfer. The increased covalency and cross-gap hybridization^30^ of transition metal 3*d* states and O 2*p* orbitals are an effective new catalyst design criteria for improving OER activity that supports oxygen evolution taking place via the LOM mechanism, a mechanism which has already been demonstrated to apply to other catalyst materials as well. Furthermore, our work illustrates the remarkable catalytic activity of the RP LSNF series that encompasses a range of chemical substitutions and electronic configurations not accessible in other crystalline metal oxide phases and that enables the elucidation of crucial structural–chemical–electronic relationships and the OER mechanism that has not been possible with Ni–M oxyhydroxides and other reported metal oxide catalysts for OER.

## Results

### Materials characterization

**Fig. 1:**
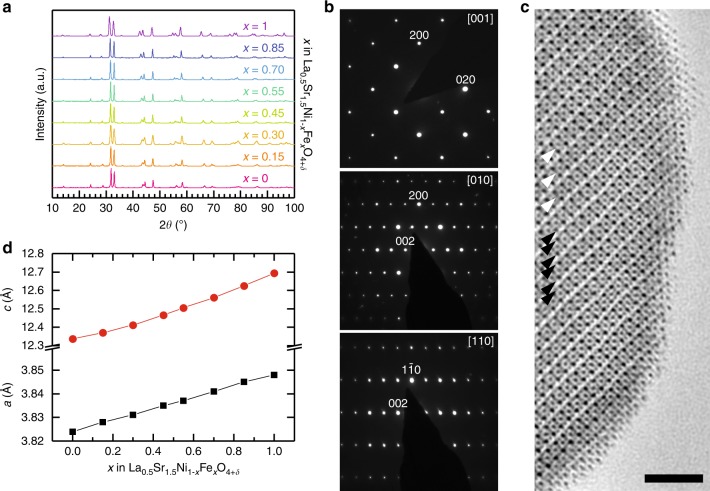
Crystallographic characterization of the La_0.5_Sr_1.5_Ni_1−*x*_Fe_*x*_O_4+*δ*_ series. **a** PXRD patterns of La_0.5_Sr_1.5_Ni_1−*x*_Fe_*x*_O_4+*δ*_ demonstrate all compositions have the tetragonal *n* = 1 Ruddlesden–Popper *I*4/*mmm* structure. **b** Electron diffraction patterns of LSNF30 confirm the absence of cation and/or anion ordering. **c** [100] ABF-STEM image of LSNF30 directly visualizes the stacking of the (BO_2_) octahedra (white arrowheads, B = Ni, Fe) and (AO)(OA) layers (black arrowheads, A = La, Sr) in the Ruddlesden–Popper structure. The scale bar is 2 nm. **d** Compositional dependence of *a* and *c* unit cell parameters showing 0 ≤ *x* ≤ 1 homogeneity range

La_0.5_Sr_1.5_Ni_1−*x*_Fe_*x*_O_4±*δ*_ (LSNF, *x* = 0 to 1) samples were synthesized using a modified Pechini method^31^ followed by crystallization and annealing, details of which are found in the Methods section. Electron diffraction and powder X-ray diffraction (PXRD) patterns correspond to a body-centered tetragonal unit cell with the *I*4/*mmm* space group, characteristic of the *n* = 1 Ruddlesden–Popper (RP) crystal structure (Figs. 1a, b and Supplementary Figure 1). No superlattice reflections were detected that could be attributed to the La/Sr or Fe/Ni ordering, ordering of hyperstoichiometric oxygen atoms, or lattice distortions. The *a* and *c* unit cell parameters (Fig. 1d) and unit cell volume (Supplementary Figure 2) increase almost linearly with Fe substitution reflecting that the La_0.5_Sr_1.5_Ni_1−*x*_Fe_*x*_O_4±*δ*_ solid solution is homogeneous over the entire substitutional range. The *I*4/*mmm* structure has been confirmed with Rietveld refinement from PXRD data (Supplementary Table 1, Supplementary Figures 3 and 4). Unit cell volume increases with Fe substitution are in agreement with the increasing fraction of Fe cations that have larger ionic radii than Ni cations (*r*(Fe^3+^, HS) = 0.645 Å, *r*(Fe^4+^, HS) = 0.585 Å, *r*(Ni^3+^, LS) = 0.56 Å, *r*(Ni^4+^, LS) = 0.48 Å)^32^. The ratio of the apical to equatorial Ni/Fe–O distances increases from 1.038 in LSN to 1.073 in LSF, reflecting slight apical elongation of the (Ni/Fe)O_6_ octahedra. The *I*4/*mmm* crystal structure is also directly viewed with annular bright field scanning transmission electron microscopy (ABF-STEM, Fig. 1c), which visualizes heavier cations and lighter oxygen anions simultaneously. The ABF-STEM image shows perfect stacking of the perovskite (BO_2_) (B = Ni, Fe) layers and the rocksalt (AO)(OA) (A = La, Sr) layers without stacking faults and the crystal structure propagates to the surface without amorphization.

The consistent morphology of catalysts is crucial to the analytical comparison of electrochemical OER activity. High-angle annular dark-field scanning TEM (HAADF-STEM) and Brunauer–Emmet–Teller (BET) surface area analysis were performed and the results, presented in Supplementary Figure 5, indicate similar morphology across the LSNF series regardless of Fe substitution. All compositions consist of 50–300 nm crystallites loosely sintered into agglomerates that are up to several microns in size. This morphological similarity is reinforced by BET surface area results which range from 3.3 to 8.0 m^2^ g^−1^ (Supplementary Figure 6).

### Electrochemistry

**Fig. 2:**
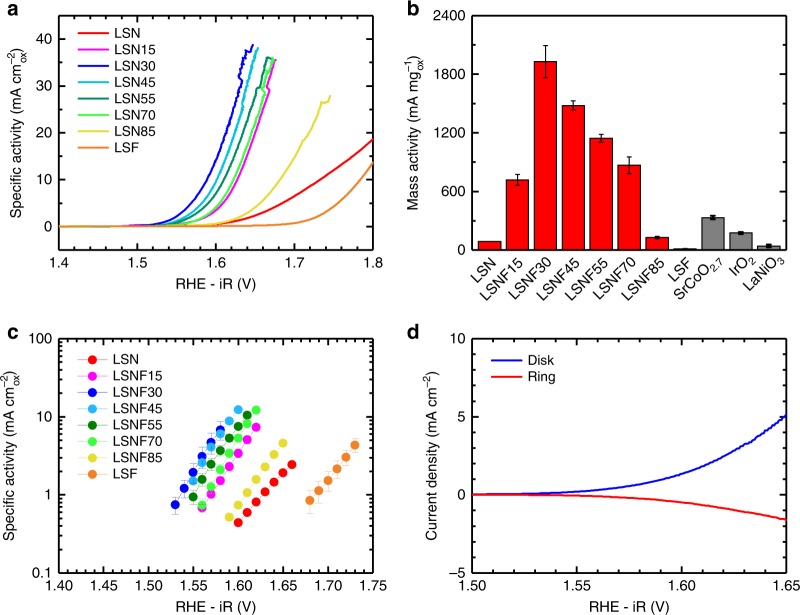
Oxygen evolution results and catalytic activities for the LSNF series supported at 30 wt% on XC72 Vulcan carbon (VC). Activities measured in O_2_-saturated 0.1 M KOH at 10 mV s^−1^ and 1600 rpm. **a** Averaged (anodic and cathodic) OER polarization curves presented in geometric current density (5 mm GCE, A = 0.196 cm^2^). **b** Oxide mass activities of the LSNF series at 1.63 V vs RHE-iR compared to leading OER catalysts SrCoO_2.7_, LaNiO_3_, and IrO_2_ on VC, all tested in the same experimental setup and conditions. Error bars are the standard deviations of measurements performed in triplicate. **c** Tafel plots of the specific activity of each LSNF catalyst. Error bars are the standard deviations of measurements performed in triplicate **d** RRDE test to confirm that current is due to oxygen evolution. The disk has a thin layer of 30 wt% LSNF30/VC and the ring is Pt. O_2_ generated at the disk is then reduced back to OH^−^ at the ring which is poised at −0.4 V vs Hg/HgO (1 M). The collection efficiency of the RRDE at 1.63 V was found to be 34%. RRDE measurements were performed in Ar-saturated 0.1 M KOH

The La_0.5_Sr_1.5_Ni_1−*x*_Fe_*x*_O_4±*δ*_ (LNSF) series was supported on Vulcan carbon (VC) at 30 wt% and tested for the OER with the results presented in Fig. 2. Supporting the catalyst on VC increases the conductivity of the composite by facilitating increased electrical contact between the glassy carbon RDE and the catalyst particles as well as between catalyst particles themselves. While carbon has its limitations as a support for device-level applications, the issue of carbon corrosion is a matter of kinetics and for fundamental studies such as this one it is quite common to support metal oxide catalysts on carbon when doing OER studies at room temperature. A major reason for this is to eliminate possible contributions from other supports, such as Ni foam, that while more appropriate for device-level mass loadings and performance can contribute a great deal to the OER current generated or have their own redox features that can convolute the examination of the material of interest and the fundamental principles explored in this work. The carbon ratio used in this paper was chosen as a result of our past work in which we carefully studied the effect of the catalyst to carbon ratio to determine that a 30:70 weight ratio resulted in optimal catalyst utilization^33^.

Representative polarization curves for all supported compositions are shown in Fig. 2a and Supplementary Figure 7. For all amounts of Fe substitution, except for 100% (LSF), the onset potential for the OER decreases and the corresponding catalytic activity increases significantly compared to LSN. Merely 15% replacement of Ni with Fe increases the specific activity (mA cm^−2^_oxide_) by over an order of magnitude at 1.63 V vs the reversible hydrogen electrode corrected for electrolyte resistance (RHE-iR) as seen in Fig. 2b. Further substitution of Fe for Ni yields a volcano-like catalytic trend with 30% Fe substitution in LSNF30 being the most active composition. LSNF30 displays exceptionally high catalytic activities of 32.7 mA cm^−2^_oxide_ and 1930 mA mg^−1^_oxide_ at 1.63 V, over 20 and 40 times higher than the respective values for LSN, with a Tafel slope of 44 mV dec^−1^ and achieves the common benchmark of 10 mA cm^−2^_geo_ at only a 360 mV overpotential which is remarkable considering the relatively small mass loading used (Fig. 2c). Details regarding conversion of potentials from Hg/HgO to RHE can be found in the Methods and Supplementary Figure 8. Figure 2b and Supplementary Figure 9 demonstrate the significantly higher mass activity of LSNF30 compared to other leading metal oxide OER catalysts with LSNF30 being over five times more active than the recently reported SrCoO_2.7_ (1930 vs 332 mA mg^−1^_oxide_) and over an order of magnitude better than IrO_2_ (173 mA mg^−1^_oxide_), a leading precious metal oxide benchmark catalyst. The LSNF30 composition generates three times as much current per surface area as SrCoO_2.7_ (32.7 vs 9.2 mA cm^−2^_oxide_) and over an order of magnitude more than IrO_2_ (1.2 mA cm^−2^_oxide_) at 1.63 V (Supplementary Table 2). The catalytic activity of VC is negligible, contributing only 7 mA mg^−1^ (Supplementary Figures 7 and 9) at 1.63 V. Cyclic voltammetry (CV) experiments using a rotating ring disk electrode (RRDE) were performed to ensure the measured currents for LSNF30VC were due to oxygen evolution and not carbon oxidation or other reactions. As can be seen in Fig. 2d, at 1.63 V, the same potential at which we base our activity measurements, the measured collection efficiency of 34% closely matches the theoretical collection efficiency. While carbon corrosion may become a factor at much higher potentials where the VC support would oxidize, at the potentials we use to examine the catalyst in this fundamental context the only current measured is due to oxygen evolution, lending further validity to the trends we observe. Additional experimental details for RRDE experiments can be found in the Methods section and Supplementary Figure 10. In the absence of the VC support, much lower activities were observed (Supplementary Figure 11) due to the lack of a carbon matrix to increase conductivity, however, the same volcano trend in activities was observed across the LSNF series and LSNF30 exhibited catalytic activity comparable to IrO_2_. Supplementary Table 2 summarizes the catalytic activities of other promising catalysts to enable comparison with LSNF30 using multiple metrics. Examined in the context of both precious and non-precious metal OER catalysts, LSNF30 is one of the most active catalysts ever reported for the OER.

**Fig. 3:**
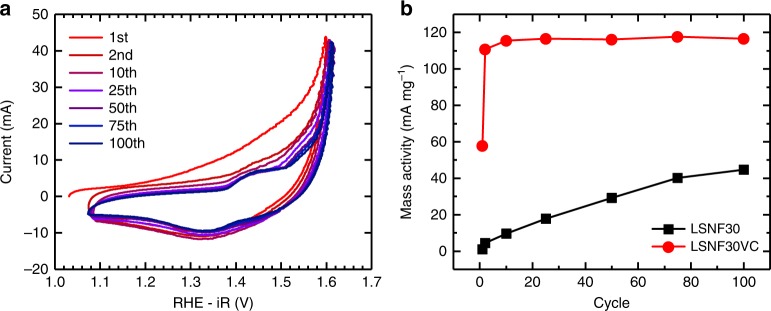
Cyclic voltammetry stability tests of LSNF30 dropcast on Ni foam electrodes at 1 mg cm^−2^. **a** One hundred cycles of LSNF30 supported at 30 wt% on VC performed in O_2_-saturated 0.1 M KOH at 10 mV s^−1^. **b** Mass activities measured at 1.63 V for both LSNF30VC and unsupported LSNF30 at various points during the 100 cycle stability tests. Mass activities were calculated by subtracting the current at 1.63 V for an Ni foam electrode with or without carbon but without catalyst from the current measured from the catalyst on Ni foam with or without the VC support and then dividing by the mass of catalyst used

The stability of LSNF30 was investigated using multiple types of electrochemical tests. One such experiment was a galvanostatic current hold during which the supported catalyst sustained 10 A g^−1^_ox_ for over 24 h without failure (Supplementary Figure 12). To further investigate the stability of the catalyst both supported and unsupported LSNF30 materials were drop cast at 1 mg cm^−2^ onto Ni foam electrodes and cycled 100 times in 0.1 M KOH. The currents measured at 1.63 V on the Ni foam that is capable of performing the OER on its own, both drop cast with VC and without, were subtracted out. As can be seen in Fig. 3 and Supplementary Figure 13 the OER current generated by the LSNF30VC composite stabilizes very quickly and displays a much higher mass activity than the unsupported catalyst due to the added conductivity of the composite that comes from the VC support. These activities are much lower than measured on RDE due to the inefficient catalyst utilization that comes from using higher mass loadings as well as mass transport limitations and the buildup of oxygen on the electrode without rotation. These stability experiments demonstrate that while a small amount of amorphization may occur at the surface during the first CV (Supplementary Figure 14) as a result of wetting or an initial restructuring such has been seen for other perovskite catalysts^34^ and as was seen for the bare Ni foam electrodes, the exceptionally high activities we observe are due to the covalency imparted by Sr substitution into the crystalline RP phase and the stability of the structure with repeated cycling. Were a transformation to amorphous oxyhydroxide phases taking place and governing the catalytic activity of the LSNF materials then we would expect much lower currents due to the decreased surface areas of active sites formed at high temperature as well as the majority of oxide mass that is made up of catalytically inactive La and Sr sites. The stability of LSNF materials was further studied using DFT and is discussed below.

### Chemical states and electronic structure

**Fig. 4:**
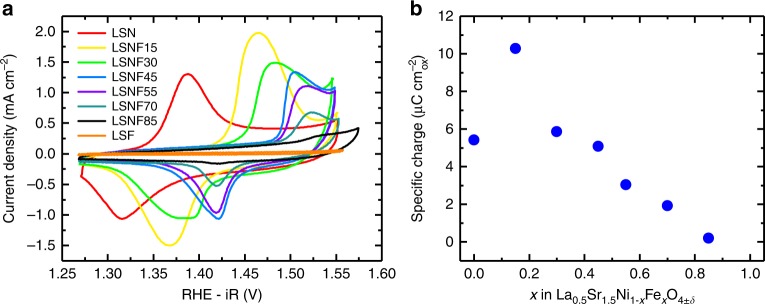
Cyclic voltammetry (CV) of LSNF catalysts in O_2_-saturated 0.1 M KOH. **a** CVs of LSNF at 100 mV s^−1^ revealing a systematic anodic shift in Ni^2+/3+^ oxidation/intercalation potentials with increasing Fe substitution. CVs at 100 mV s^−1^, taken after 3–4 cycles, are depicted to aid the reader in distinguishing peak potential shifts and relative areas, but at 100 mV s^−1^ contributions to *E*_P_ from capacitance and mass transport resistance cannot be ignored. To address this, peak potentials reported in Supplementary Figure 8c were taken from stable CVs at 10 mV s^−1^. **b** Specific oxidative charge (µC cm^−2^_oxide_) resulting from the integration of oxidation waves. Integrations were performed using specific current density (µA cm^−2^_oxide_) to normalize differences in catalyst surface area (Supplementary Figure 8d). The OER baselines for stable CVs at 10 mV s^−1^ were fit and subtracted. Supplementary Figure 14 contains the CVs used for integration

Upon exposure to alkaline electrolyte the surface of the catalyst is hydroxylated and reduced. The Ni^2+/3+^ redox couple in LSNF is observed to shift to more positive potentials with Fe substitution, indicating a direct modulation of Ni’s reactivity with Fe substitution. This redox couple exists in the incipient OER region, as indicated in the reversible CV peaks in Fig. 4a. The potential range over which the redox peaks are observed is consistent with prior studies of Ni-based electrocatalysts^3,35,36^ with Fe substitution shifting the peak potential (*E*_P_) of Ni^2+/3+^ oxidation as documented with Ni–Fe oxyhydroxides^3,4,35^. Integration of the oxidation waves (Supplementary Figures 15 and 16) reveals that the specific oxidative charge (µC cm^−2^_oxide_) transferred during oxidation/intercalation consistently decreases upon continued replacement of Ni with Fe, with the exception of the initial introduction of Fe in LSNF15 (Fig. 4b). Similar behavior was previously reported for 10% Fe substitution into NiOOH hydroxide electrodes^3^ and was speculated to be due to increased oxygen and electrolyte diffusivity. This interpretation is supported here by the increased electrochemical oxygen diffusion rate measured in LSNF15 as compared to LSN (1.04E^−12^ cm^2^ s^−1^ vs 8.03E^−13^ cm^2^ s^−1^; Supplementary Figure 17 and Supplementary Table 3) and confirms that along with the surface redox of Ni^2+/3+^, oxygen intercalation concomitant with Ni redox is likely given the labile nature of oxygen in La_2_NiO_4±*δ*_. The observation that the pH dependence of the Ni^2+/3+^ oxidation *E*_p_ in Supplementary Figure 14 behaves like a Nernstian pseudocapacitor using OH^−^ as the intercalating ion further indicates that the intercalation of oxygen is taking place^14,33,37,38^.

It is known that over 50% Sr substitution for La induces metal-like conductivity^25,39^ in the charge transfer insulator La_2_NiO_4±*δ*_ due to hole doping in the O 2*p* band^40^. Furthermore, the remarkable improvement of the catalytic activity of LSN upon Fe substitution for Ni may be explained by the impact of the Fe 3*d* states on the electronic structure of LSNF. Earlier studies of the electronic structure of La_1.1_Sr_0.9_Ni_0.8_Fe_0.2_O_4±*δ*_ with resonant photoemission spectroscopy suggest that the Fe 3*d* states make a substantial contribution to the valence band near the Fermi level (*E*_F_) by strongly hybridizing with the O 2*p* and Ni 3*d* states which facilitates cation oxidation and redox processes^28^. The mixed Fe 3*d* and Ni 3*d* bands pinned at *E*_F_ in close proximity to the O 2*p* band should give rise to a mixture of Ni^3+/4+^ and Fe^3+/4+^ where the relative proportion of oxidation states changes with the amount of Fe substitution^27,29,41^. This was confirmed by ex situ room temperature Mössbauer spectroscopy (Supplementary Figures 18 and 19, Table 1) in conjunction with iodometric titrations. Knowing the average B-site oxidation state as well as the oxidation state of Fe allows for the calculation of the Ni oxidation state and Table 1 contains the results of this analysis. The average oxidation state of Ni increases with increasing Fe content from +3.54 in LSN to +3.95 in LSN85, as does the oxygen hyperstoichiometry (*δ*) and the average B-site oxidation state. The increase in the oxidation state of Ni is further demonstrated by the observation that the Ni 3*p* spectrum obtained by X-ray photoelectron spectroscopy (XPS) shifts to higher binding energies with increasing Fe content (Supplementary Figure 20), and again by the positive shift in potential of the Ni^2+/3+^ redox features upon Fe substitution (Fig. 4a). Fitting parameters for Mössbauer spectroscopy can be found in Supplementary Tables 4 and 5. Additional discussion of oxygen stoichiometry and LSNF oxidation states can be found in Supplementary Note 1.

**Table 1:** Oxygen hyperstoichiometry (*δ*) and B-site oxidation states in La_0.5_Sr_1.5_Ni_1−*x*_Fe_*x*_O_4±*δ*_

LSNF series oxidation state characterization									
Catalyst	Fe sub.	*δ*, hyperst.	*δ* s.d.	B^+^ avg	B^+^ s.d.	Fe^4+^ (Moss.)	Fe^3+^ (Moss.)	Ni^*x*+^ avg	Ni^*x*+^ s.d.
LSN	0%	0.018	0.013	3.54	0.03	–	–	3.54	0.03
LSNF15	15%	0.046	0.007	3.59	0.01	73%	27%	3.57	0.02
LSNF30	30%	0.042	0.010	3.58	0.02	62%	38%	3.57	0.03
LSNF45	45%	0.065	0.027	3.63	0.05	57%	43%	3.68	0.10
LSNF55	55%	0.088	0.011	3.68	0.02	58%	42%	3.80	0.05
LSNF70	70%	0.076	0.022	3.65	0.04	58%	42%	3.82	0.14
LSNF85	85%	0.081	0.036	3.66	0.07	61%	39%	3.95	0.48
LSF	100%	0.143	0.037	3.79	0.07	62%	38%	–	–

Average B-site oxidation states determined by iodometry, the results of Mössbauer spectroscopy, and the calculated average Ni oxidation state

Supplementary Figures 18 and 19 contain the deconvoluted Mössbauer spectra

To further understand the electronic structure evolution across the LSNF series and its implications for the OER activity, as well as confirm the results of the Mössbauer spectroscopy and iodometric titrations, we model the bulk phase of LSNF and a simplified Sr_2_Ni_1−*x*_Fe_*x*_O_4_ (SNF) series by DFT. While the SNF system was not investigated experimentally, it was modeled to confirm that the same trends in electronic structure evolution that occur in LSNF also take place in a sample with a different A-site composition. Additionally, modeling the SNF series also eliminates a possible source of variation between the atomic arrangements of A-site elements in the modeled LSNF system vs the arrangements in the synthesized samples and verifies our results on a simplified catalyst system.

**Fig. 5:**
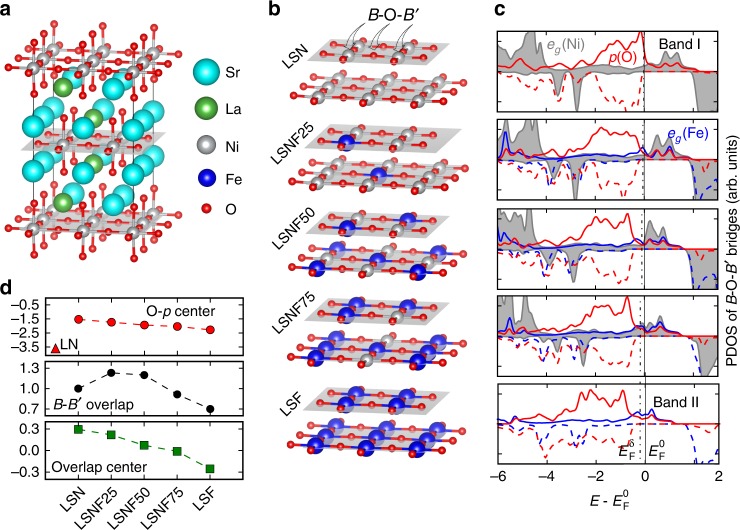
Density functional theory modeling of atomic and electronic structures of bulk LSNF in ferromagnetic configuration. **a** The ground state 2 × 2 × 1 cell of La_0.5_Sr_1.5_NiO_4_, with uniformly distributed La in each [001] *A*O layer. **b** Representations of the two *B*(*B*′)O_2_ layers for each ground state La_0.5_Sr_1.5_Ni_1−*x*_Fe_*x*_O_4_, with *x* at 0, 0.25, 0.5, 0.75, and 1; for each *x*, the ground state Fe arrangement is characterized by equal numbers of Fe in the two *B*(*B*′)O_2_ layers, with the Fe in each layer arranged to maximize the number of Fe–O–Ni bridges. **c** The corresponding spin polarized PDOS of *e*_*g*_ (Ni: gray shaded area and Fe: blue line) and 2*p* (O: red line) with respect to Fermi level ($E_{\mathrm {F}}^0$) for the *B*–O–*B′* bridges; the PDOS are the average of existing *B*–O–*B′* bridges; the adapted Fermi level ($E_{\mathrm {F}}^\delta$) to oxygen hyperstoichiometry is estimated via the rigid band model, with $E_{\mathrm {F}}^\delta = E_{\mathrm {F}}^0 - 2e^ - {\mathrm{\delta }}/{\mathrm{DOS}}(E_{\mathrm {F}}^0)$ (Eq. (1)), where ${\mathrm{DOS}}(E_{\mathrm {F}}^0)$ is the total density of states at $E_{\mathrm {F}}^0$ per formula unit of LSNF. **d** Top: Computed values of the O–*p* band center (top panel), with La_2_NiO_4_ calculated as reference. Middle: The magnitude of band overlap, determined by the integration of maximum PDOS between *e*_*g*_(*B*) and *e*_*g*_(*B*′) from −2 to 2 eV, normalized to *e*_*g*_(Ni) in LSN. Bottom: The corresponding overlap center (centroid) of maximum PDOS between *e*_*g*_(*B*) and *e*_*g*_(*B*′). Additional details can be found in Supplementary Note 2

A series of LSNF compositions are modeled by 2 × 2 × 1 primitive unit cells, allowing for unit compositions of La_0.5_Sr_1.5_NiO_4_ (LSN), La_0.5_Sr_1.5_Ni_0.75_Fe_0.25_O_4_ (LSNF25), LSNF50, LSNF75, and LSF. The SNF series was modeled using the same Ni and Fe ratios, and details regarding cell formation and magnetic investigation are given in the Methods and Supplementary Note 2. Figure 5a, b shows the (001) atomic layers of the most stable LSNF structures based on our screening. First, we look at LSN (Fig. 4a) and we find a uniform, proportional distribution of La/Sr^42^. Using this La/Sr distribution we determine the minimum energy Ni/Fe ordering which results in two key features governing the Fe arrangement in LSNF (Fig. 5b). First, each of the two *B*(*B*′)O_2_ layers has an equal number of Fe cations in order to prevent an unbalanced charge distribution between them. Second, the Fe cations in each *B*(*B*′)O_2_ plane are distributed such that the number of Fe–O–Ni bridges is maximized^2^. This arrangement mitigates the effects of the induced O 2*p* electron hole at Ni–O–Ni bridges due to more effective electron donation by Fe at Fe–O–Ni bridges, which increases stability^18^. Compared with Fe–O–Fe bridges, the Fe–O–Ni bridges induce shorter Fe–O bond lengths and indicate higher Fe oxidation states, a trend that also occurs in the simplified SNF system. This observation agrees with the experimentally observed predominance of Fe^4+^ over Fe^3+^ at low Fe substitution as well as the increase in unit cell parameters as Fe content increases (Fig. 1d, Supplementary Table 1).

The computed projected density of states (PDOS) of the Fe–O–Ni bridges in both LSNF and SNF confirm that the Ni cations are in the low-spin state with fully occupied $t_{2g}^ \downarrow$ states (Supplementary Figures 21–23) while the Fe cations are in the high-spin state with mostly unoccupied $t_{2g}^ \downarrow$ states for all LSNF and SNF^43^. These spin states lead to *e*_*g*_ bands positioned around the Fermi level $(E_{\mathrm {F}}^0)$, thus becoming the most relevant for Fe–O–Ni interactions of interest and the focus of our study. Inspection of the Fe–O–Ni bridges reveals significant overlap of O 2*p*, Ni $e_g^ \uparrow$ and Fe $e_g^ \uparrow$ bands for all compositions in the LSNF series (Fig. 5c), in agreement with our predictions. The same overlap is observed in the SNF series with only a small downward shift in *E*_F_ due to increased oxidation of the B-site elements to differentiate the two (Supplementary Figure 23). This overlap leads to two important observations. First, the unoccupied O 2$p^ \uparrow$ states indicate oxygen electron holes with finite PDOS across $E_{\mathrm {F}}^0$, thus bridging charge transfer between neighboring cations^32,43^. Second, the similar energies of the Ni and Fe $e_g^ \uparrow$ states (denoted Band I and Band II in the following) open up the possibility of electron exchange through oxygen. These two factors govern the evolution of the PDOS from LSN to LSF and from SN to SF. On the one hand, the substitution of Ni by less electronegative Fe stabilizes O 2*p* electrons and increases the Ni–O distances, shifting the O 2*p* band center downwards relative to *E*_F_ (Fig. 5d, Supplementary Figure 23) and decreasing the bandwidth^43^ which reduces electron hole concentration and charge transfer ability^44^. This condition is consistent with the experimentally observed increase in oxygen hyperstoichiometry going from LSN to LSF (Table 1, Supplementary Table 3). On the other hand, the partially filled Band II (Fe) significantly hybridizes with both O 2*p* and Band I (Ni), driving the latter towards $E_{\mathrm {F}}^0$. Both Bands I and II make significant contributions to the occupied (valence) and unoccupied (conduction) states, respectively, and this dramatically increases the bandwidth of the triple band overlap going from LSN to LSNF50 and from SN to SNF50, with its center approaching $E_{\mathrm {F}}^0$. As the *e*_*g*_ states are relevant for surface chemisorption, the increasing bandwidth around $E_{\mathrm {F}}^0$ would decrease the energetic cost to accept/donate electrons^28^ at the adsorbate-catalyst interface, and therefore should increase the rate of reaction according the Gerischer–Marcus model of charge transfer^45^. The increased Fe concentrations in LSNF75 and SNF75, however, drive Band I (Ni) into the similar distribution of Band II (Fe) and this reduces the bandwidth (Fig. 5d). This suggests that the optimal OER activity will occur for a composition between LSNF25 and LSNF50, consistent with experimental observations. Although these calculations are performed for the bulk structure of LSNF, the surface electronic structure is expected to be strongly correlated to that of bulk, similar to *AB*O_3_ perovskites as was demonstrated in previous work^46^, thus preserving the key feature of Ni–O–Fe hybridization and its evolution across LSNF and SNF compositions.

## Discussion

The computed band structure of LSNF is similar to that reported for other highly active OER catalysts, especially those compositions with mixed transition metal sites such as Ni–Fe layered double hydroxide catalysts^2^. Conesa calculated the DOS of one such polymorph, FeNi_7_O_8_(OH)_8_ in the 2H_C_ structure with Fe in O_4_(OH)_2_ coordination, and their result is strikingly similar to that of LSNF in that Ni and Fe 3*d* bands are strongly hybridized just above *E*_F_. They concluded that Fe^4+^ is stabilized by induced charge transfer between Ni sites, which is also seen in Ni–Fe oxyhydroxides^6^, and this explanation agrees with our observation of Fe in the 4+ oxidation state in LSNF (Table 1). The shifting of the Ni^2+/3+^ redox couple upon Fe substitution that we observe (Fig. 4) has previously been reported in NiOOH upon incorporation of Fe and is further evidence of this partial charge transfer between Fe and/or Ni^4,5,47^. A change in covalency by the introduction of FeO_6_ units into LSN must have an inductive effect on Ni through next nearest neighbor interactions via Fe–O–Ni bridges^48^, which is consistent with the observation that our activity is maximized for the composition having the highest probability of Fe–O–Ni bridging interactions, LSNF30.

The band structure of LSNF also resembles the computed band structures of LaCo_1−*x*_Fe_*x*_O_3_(ref.^49^), Sr_2−*x*_La_*x*_MO_4±*δ*_(ref.^50^), and Ba_0.5_Sr_0.5_Co_0.75_Fe_0.25_O_3±*δ*_ (BSCF)^51^. Merkle calculated the band diagram for BSCF and found a similar electronic configuration as in LSNF, and Schmidt and co-workers saw that the catalytic activity of Ba_0.5_Sr_0.5_Co_0.8_Fe_0.2_O_3±*δ*_ was much higher than that of Ba_0.5_Sr_0.5_CoO_3±*δ*_(ref.^34^). Thus it is possible that the high activity of BSCF, SrNb_0.1_Co_0.7_Fe_0.2_O_3−*δ*_^34,52^ as well as others with similar mixed transition metal sites may be rationalized not just by the covalent bonding, but also by triply-overlapping Co/Ni and Fe 3*d* states with O 2*p* near *E*_F_ similar to the LSNF series, which has largely been overlooked up to now. Furthermore, the similarities between the band structures of LSNF materials and these other active catalysts of varying structures, some of them amorphous, reinforces how the principle of cross-gap hybridization may apply to many different metal oxide catalysts and applies to the LSNF series even in the event of surface restructuring.

As we have discussed in our previous work, the OER can proceed by the adsorbate exchange mechanism (AEM) or the LOM mechanism^14,18^. In the AEM, chemisorbed intermediates undergo a series of electrochemical oxidations as the transition metal active site undergoes oxidation and reduction, and these redox reactions are the most energetically intensive and thus rate limiting steps^14^. The LOM OER mechanism, on the other hand, does not require significant redox switching of transition metal sites but instead requires the participation of lattice oxygen in the OER. Ligand holes that arise when transition metal 3*d* bands are highly covalent with O 2*p* bands and exist around $E_{\mathrm {F}}^0$ activate lattice oxygen that combines with chemisorbed OH to produce O_2_^−^. In previous studies we found that the DFT-computed O_vac_ formation energy in bulk *AB*O_3_ perovskites (donated as $\Delta E_{V_O}$) served as an effective indicator of OER mechanism^18^. Highly covalent perovskites favor O_vac_ formation that results from an overall high-energy lying O 2*p* band relative to $E_{\mathrm {F}}^0$ and the appearance of electron holes that facilitate reversible lattice oxygen participation under OER conditions. Employing $\Delta E_{V_O}$, we showed that the mechanism for oxygen evolution switches from the AEM to the LOM with decreasing $\Delta E_{V_O}$ for the La_*x*_Sr_1−*x*_CoO_3−*δ*_ series when *x* > 0.4 (ref.^14^). The decrease in oxygen vacancy formation energy in these materials occurred in concert with an increase in oxygen diffusion rates and OER activity^14^. This proposed mechanistic switch was experimentally confirmed by a recent report from the Shao Horn group who used isotopic labeling of lattice oxygen to observe a transition from the AEM on LaCoO_3_ to the LOM on La_0.5_Sr_0.5_CoO_3−*δ*_ and SrCoO_3−*δ*_^17^.

The same type of band overlap and resulting covalency that is a requirement of the LOM mechanism and that occurs in the previously reported materials discussed above also occurs in LSNF. The hybridization of *e*_*g*_(Ni) and *e*_*g*_(Fe) bands with the *p*(O) band across $E_{\mathrm {F}}^0$ results in increased oxygen electron holes, and DFT computations reveal that $\Delta E_{V_O}$ in LSN, LSNF25, and LSNF50 at Ni–O–Fe bridges is more negative than in LaNiO_3_ and La_0.5_Sr_0.5_CoO_3−*δ*_^18^ while $\Delta E_{V_O}$ in LSNF75 and LSF is larger (Supplementary Table 6). Thus $\Delta E_{V_O}$, a proven indicator of the LOM mechanism, works in conjunction with the experimentally measured increased oxygen diffusion rates from LSN to LSNF45 (Supplementary Figure 17, Supplementary Table 3), to suggest that the exchange of surface lattice O plays a key role in OER and that the highly active LSNF materials utilize a LOM-type mechanism. The catalytic effect from the LOM mechanism is further enhanced by the substitution of Fe that causes broadening of *e*_*g*_ bandwidth through Ni–O–Fe cross-gap hybridization. Altogether this demonstrates how the LOM applies to a new series of materials and solidifies the importance of both increased covalency that results in oxygen electron holes, as well as the cross-gap hybridization that causes broadening of the overall bandwidth near *E*_F_ in the design and optimization of oxide catalysts.

We have precisely synthesized a series of RP catalysts having highly oxidized and covalent Ni 3*d*–O 2*p*–Fe 3*d* bonds that give rise to exceptional OER activity. Sr substitution into the perovskite LaNiO_3_ has previously been proposed as an avenue to increase the oxidation state if Ni and Ni–O covalency but has not been fully realized. In this work we were able to avoid inactive secondary phases resulting from poor solubility of Sr in the perovskite phase through utilization of the RP crystal structure. Additionally, by using La_0.5_Sr_1.5_NiO_4±*δ*_ as the host lattice we achieved complete substitution of Fe for Ni across the entire compositional range. The crystalline RP structure and the high degree of phase purity enabled precise study of the impact of Fe substitution on the chemical and electronic properties of the LSNF series, something that has not been possible up to this point with other, amorphous materials. Iodometric titrations coupled with Mössbauer spectroscopy indicate that the average Ni oxidation state in the LSNF series increases from +3.46 to +3.95 with increasing Fe substitution while the Ni^2+/3+^ redox peaks also shift to more positive potentials, consistent with Ni developing a more oxidized character. The influence of Fe substitution extends beyond increasing the oxidative strength of Ni, however, as the electrocatalytic activity increases by over an order of magnitude from LSN to LSNF30 despite possessing statistically equivalent Ni oxidation states. DFT calculations reveal that Fe substitution results in cross-gap hybridization where the Fe 3*d e*_*g*_ band is hybridized with both the Ni 3*d e*_*g*_ and the top of the O 2*p* density of states across the Fermi level. The increased covalency of the Ni–O bonds as well as facile charge transfer through Fe–O–Ni bridges due to incorporation of Fe explains the enhanced catalytic activity going from LSN to LSNF30. Furthermore, the increased covalency demonstrated via calculated partial density of states for LSNF and a simplified SNF that is a requirement for the OER to take place via a LOM mechanism resembles the band structure of other highly active OER catalysts with varying crystal or amorphous structures for which this mechanism has already been verified. Decreased oxygen vacancy formation energies and increased rates of oxygen diffusion, descriptors already accepted in the literature as indications of the LOM, are seen on LSNF and further support the hypothesis that the OER proceeds via a LOM mechanism on LSNF. This methodology of selective A- and B-site substitution to promote cross-gap hybridization in RP oxides reveals important fundamental aspects related to their structure and the exceptional electrocatalytic activities of these materials as well as other metal oxide catalysts.

## Methods

### General

All chemicals were used as they were received from manufacturers. Lanthanum(III) nitrate hexahydrate (99.995%), strontium(II) nitrate hexahydrate (99%), nickel(II) nitrate hexahydrate (99%), iron(III) nitrate non-ahydrate (99.99%), tetramethylammonium hydroxide pentahydrate (TMAOH, 99%), diethylene glycol (DEG, 99.99%), citric acid monohydrate, 2-propanol, ethylenediaminetetraacetic acid, and potassium hydroxide (KOH) were purchased from Fisher Scientific. Absolute 200 proof ethanol was obtained from Aaper Alcohol. 5 wt % Nafion solution in lower alcohols and IrO_2_ powder were obtained from Sigma-Aldrich. Millipore high-purity water (DI water, 18 MΩ) was used in all experiments and synthesis and research grade, 99.999% purity oxygen and argon gases were purchased from Praxair. Vulcan XC72 carbon (VC) was purchased from Cabot Corporation and ball-milled prior to use.

### Catalyst synthesis

La_0.5_Sr_1.5_Ni_1−*x*_Fe_*x*_O_4±*δ*_ (LSNF, *x* = 0 to 1) samples were synthesized using a modified Pechini method^29^ followed by crystallization and annealing. A- and B-site nitrate salts, in stoichiometric ratios, were dissolved in water to create a solution in which the total concentration of metal salts was 0.1 M. Citric acid and EDTA were added, each at a concentration of 0.1 M, and then 1 M TMAOH was added dropwise until the pH had reached 7.5 in order to deprotonate and dissolve the EDTA. DEG was then injected to reach a concentration of 0.067 M and the solution was heated to 85 °C while stirring. The EDTA and citric acid were both added to ensure complete chelation of the metal cations, preventing agglomerations or particle formations that may lead to catalyst inhomogeneity. DEG and heat were added to the solution to drive a dehydration reaction between the polyhydroxyl alcohol and the carboxylic acid groups of the chelates to form a polyester gel. Once all the water had been evaporated the gel was combusted on a hot plate at 350 °C to form mixed metal oxide precursor particles. This step was performed on a hot place and not in a sealed tube furnace to avoid possible explosions from rapid evolution of gasses upon combustion. Finally, precursor particles were crystallized at 950 °C (heated at 20 °C min^−1^) for 5 h_,_ then cooled to 400 °C and left to anneal for 6 h in a tube furnace. The entire crystallization and annealing routine was done under pure O_2_ flowing at 200 mL min^−1^. Catalysts were recovered and immediately stored under Ar gas to prevent catalyst surface amorphization. Total catalyst yields per synthesis ranged from 500 to 750 mg. More details regarding the synthesis can be found in Kakihana et al.^31^

SrCoO_2.7_ and LaNiO_3_ were synthesized via a coprecipitation method previously reported elsewhere^13,46^ in which A and B-site nitrate salts in a 1:1 ratio were dissolved in a 1 wt% solution of TMAOH containing an equimolar amount of TPAB to the total moles of metal cations in order to form mixed metal hydroxide precursor particles. These solutions were flash-frozen on a rotating metal drum that had been cooled to cryogenic temperatures and was collected before lyophilization to remove water. The precursor particles were then calcined at 700 °C (LaNiO_3_) or 950 °C (SrCoO_2.7_) to form the perovskite phase.

### Electrochemical characterization

To make catalyst inks 2 mg of catalyst powder were added to 2 mL of NaOH neutralized 0.05 wt% Nafion solution^47^ and bath sonicated for at least 1 h. Ten microliters of catalyst ink was drop cast onto 5 mm glassy carbon electrodes (GCE) from Pine Instruments and dried in air overnight. All GCEs were polished before drop casting via sonication in a solution of equal parts water and ethanol followed by polishing using 0.05 μm alumina powder after which they were again sonicated in a fresh solution of water and ethanol and dried in ambient air. This method was used to clean all GCEs prior to drop casting, after which a composite catalyst loading of 51 μg_total_ cm^−2^_geo_, yielding 15.3 μg_oxide_ cm^−2^_geo_ was achieved for catalysis and intercalation tests.

CH Instruments CHI832a or Metrohm Autolab PGSTAT302N potentiostats were used to carry out all electrochemical testing. Potentiostats were equipped with high-speed rotators from Pine Instruments. Experiments were performed at room temperature in 0.1 M KOH (measured pH ≈ 12.8). Electrolyte resistance was measured using current interrupt and positive feedback methods (46 Ω) and iR compensation was applied to all electrochemical measurements after testing unless otherwise noted. A 3 electrode cell was used for all experiments. The reference electrode was a CH Instruments Hg/HgO (1 M KOH) reference electrode, a Au wire served as the counter electrode, and GCEs drop cast with catalyst ink and dried as described above were used as the working electrode. All potentials were measured vs Hg/HgO but were reported vs the reversible hydrogen electrode (RHE), which was determined experimentally to be +0.8976 V vs. Hg/HgO (1 M KOH). Supplementary Figure 8 contains the RHE calibration data.

All testing for OER activities was performed on newly dropcast electrodes which had not undergone previous testing, dropcast with 30 wt% catalyst on VC (15.3 μg_oxide_ cm^−2^_geo_) or unsupported catalyst. CV scans were performed from 1 to 2 V vs RHE at a scan rate of 10 mV s^−1^ while rotating at 1600 rpm in O_2_-saturated 0.1 M KOH. The anodic and cathodic scans were averaged and iR corrected, and the current at 1.63 V vs RHE-iR was used for comparison of OER activities. Scatter in the data at high current densities is due to oxygen bubble formation and desorption on the electrode surface. Data presented in this report are the average of at least three tests on fresh electrodes. OER testing using Ni foam supports were performed under the same electrolyte conditions but dropcast at 1 mg cm^−2^ and held stationary.

All CVs were taken on fresh electrodes of 30 wt% oxide on VC. All catalysts were first conditioned by performing 20 cycles from 1.26 to 1.55 V vs RHE at 100 mV s^−1^. Immediately following, three cycles at 100, 50, 25, 10, and 5 mV s^−1^ were collected. Unless otherwise stated, all cycles shown are the third cycle at a given scan rate. For samples containing more than 50% Fe substitution, it was necessary to perform four cycles at each scan rate. Additionally, LSNF85 had to be cycled to 1.575 V before any oxidative features were observed.

For comparison of charge passed for all oxidative waves, the last cycle at 10 mV s^−1^ was used to compute specific current density. The baseline of each CV was fit and subtracted from the specific current density. The background subtracted CVs (Supplementary Figure 16) were then integrated to find the total charge transferred.

All RRDE tests were performed in argon-saturated, 0.1 M KOH on fresh 30 wt% oxide films. LSNF30 was cycled at 10 mV s^−1^ on the disk while the Pt ring was held at −0.4 V vs. RHE. One-fourth the normal geometric loading of catalyst was used (3.8 μg_oxide_ cm^−2^_geo_) to limit the amount of oxygen generated a minimize bubble formation which reduces collection efficiency. Additional information regarding the RRDE calibration data can be found in Supplementary Figure 10.

All oxygen diffusion tests were performed in argon-saturated, 1 M KOH on fresh 85 wt% oxide films. Each LSNF composition was cycled at 20 mV s^−1^ until the oxidative peak potential did not change upon further cycling. Chronoamperometry was then performed at potentials 10 mV more anodic than *E*_p_ to ensure diffusion-limited intercalation. Linear regression was performed as described elsewhere^48,49^ to determine the diffusion rate. Particle sizes were estimated using the surface area calculated from BET measurements and densities determined by Rietveld analysis. All values reported are the average of at least three tests.

### Iodometric titrations

Iodometric titrations were performed following the procedure referenced above^13^. In this procedure 3 mL of 2 M KI solution that had previously been deoxygenated were combined in a flask with 15–20 mg of perovskite and stirred for 3 min under argon, after which 25 mL of 1 M HCl was added to dissolve the perovskite. The solution was then titrated with a solution of ~26 mM solution of Na_2_S_2_O_3_ that had been pre-standardized with 0.1 N KIO_3_ until a faint golden color was reached, after which a starch indicator was injected and the titration was continued until the solution was clear, marking the end point.

### Catalyst preparation and support

Fresh, Ar-sealed catalysts were ball milled for 3 min using a Wig-L-Bug spectroscopic grinding mill. To support the catalysts onto VC, the correct amounts of pre-ground catalyst and pre-ground VC were measured into the grinding mill’s vial and ball milled for 3 min.

### Powder X-ray diffraction (PXRD)

Catalyst structure was examined by performing X-ray diffraction with a Rigaku MiniFlex600 Diffractometer. Measurements were performed at 298 K in ambient conditions while the instrument operated at 40 kV and 15 mA using Cu Kα radiation (1.54 Å wavelength). For all tests, argon-sealed catalyst powder was exposed to ambient air and scanned over 10–100° 2*θ* in 0.01° increments with a dwell time of 0.35 s per step. PXRD patterns for the Rietveld refinement were taken with a Huber G670 Guinier diffractometer (Cu K_α1_ radiation; curved Ge(111) monochromator; image plate). The refinement was performed with the JANA2006 package^50^. As the electron diffraction patterns of all compositions revealed no deviation from the RP *n* = 1 *I*4/*mmm* structure, the La_2_NiO_4_ structure^51^ was used as a starting model. The La/Sr occupancy factors of the A positions were refined; the Fe/Ni occupancy factors for the B positions were assigned according to the results of the EDX analysis. The crystallographic data, positional and atomic displacement parameters, interatomic distances, and reliability factors are listed in Supplementary Table 1.

### Surface area analysis

Catalyst surface areas were measured via nitrogen sorption analysis which was performed at a temperature of 77 K on a Quantachrome Instruments NOVA 2000 high-speed surface area BET analyzer. Before BET experiments were performed, all catalyst materials were ball milled for 3 min followed by degassing under vacuum at room temperature for at least 12 h. Only data in the relative pressure range (*P*/*P*0) of 0.05 to 0.30, with a minimum *R*^2^ of 0.995 and *C* value of 20 was used to calculate the specific surface area using the BET method.

### Transmission electron microscopy

Preparation of TEM samples was performed by mixing the crystals with ethanol and crushing them in a mortar, after which drops of the suspension were deposited onto holey carbon grids. Electron diffraction patterns, TEM images, HAADF-STEM images, ABF-STEM images, and energy dispersive X-ray (EDX) spectra were collected using an aberration-corrected Titan G^3^ electron microscope equipped with a Super-X EDX system, operating at 200 kV using a convergence semi-angle of 21.6 mrad.

### Mössbauer spectroscopy

^57^Fe Mössbauer spectra were collected using a constant acceleration spectrometer MS1104 (Rostov-na-Donu, RF) in transmission mode with a ^57^Co/Rh γ-ray source. Calibration of the spectrometer was performed using standard sodium nitroprusside absorbers. All isomer shift values (IS) are referred to α-Fe at room temperature and spectra evaluation was performed using “UnivemMS” (Rostov-na-Donu, RF) and custom least squares fitting software with Lorentzian–Gaussian line shapes. Details regarding the deconvolution of the spectra are found in the text accompanying Supplementary Figure 18.

### DFT modeling

The Vienna Ab initio Simulation Package (VASP) was used to do spin polarized calculations^52^, using the PAW pseudopotentials and the exchange-correlation functional of Perdew–Burke–Ernzerhof (PBE), with the effective Hubbard *U*_eff_ of 5.3 (Fe) and 6.2 eV (Ni), respectively. The Ruddlesden–Popper bulk phase is modeled by fully relaxing a 2 × 2 × 1 primitive unit cell, La(Sr)_16_Ni(Fe)_8_O_32_, with a plane wave cutoff energy of 520 eV, Monkhorst-Pack *k*-point of 4 × 4 × 2, forces convergence criterion of 10–4 eV Å^−1^ and Gaussian smearing, where the smearing width is 0.1 eV. All models follow ferromagnetic ordering, as our calculations indicate its lower energy than those of nonmagnetic and antiferromagnetic orderings (A, C, and G type). Details regarding the determinations of effective Hubbard *U*_eff_, atomic, magnetic and electronic structures, and oxygen hyperstoichiometry effects are provided Supplementary Note 2.

### X-ray photoelectron spectroscopy

Characterization of the chemical states was done using a Kratos AXIS Ultra DLD XPS and a monochromatic Al X-ray source (Al α, 1.4866 eV), scanning in 0.1 eV steps with a dwell time of 4 s per step. Charge compensation was used for all samples. Binding energies were for all spectra were calibrated against the adventitious carbon peak at 285 eV. CasaXPS was used for all data analysis and deconvolution. Details regarding the deconvolution of the Ni 3*p* spectrum are found in the text accompanying Supplementary Figure 20.

### Determination of Ni and Fe oxidation states

Together, the iodometric titrations and the Mössbauer spectroscopy enable the calculation of Ni’s average oxidation state and the relative percentage of Fe^3+/4+^. Supplementary Figure 18 contains the deconvolution method and Mössbauer spectra.

### Data availability

The authors declare that all data collected and discussed in support of the findings of this study are available within the paper and its Supplementary Information.

## Electronic supplementary material

Supplementary Information
