# Supplementary material for: Exceptional electrocatalytic oxygen evolution via tunable charge transfer interactions in La0.5Sr1.5Ni1−xFexO4±δ Ruddlesden-Popper oxides
Source: Nat Commun. 2018 Aug 8;9:3150. doi: 10.1038/s41467-018-05600-y (PMC6082882; doi:10.1038/s41467-018-05600-y)
Supplement: Supplementary file 1 — Supplementary Information [file 41467_2018_5600_MOESM1_ESM.pdf]

Supplementary Information for

Exceptional Electrocatalytic Oxygen Evolution Via Tunable Charge Transfer Interactions in $La_{0.5}Sr_{1.5}Ni_{1-x}Fe_xO_{4\pm\delta}$ Ruddlesden-Popper Oxides

Robin P. Forslund[†], William G. Hardin[†], Xi Rong, Artem M. Abakumov, Dmitry Filimonov, Caleb T. Alexander, J. Tyler Mefford, Hrishikesh Iyer, Alexie M. Kolpak, Keith P. Johnston & Keith J. Stevenson

[†] These authors contributed equally to this work.

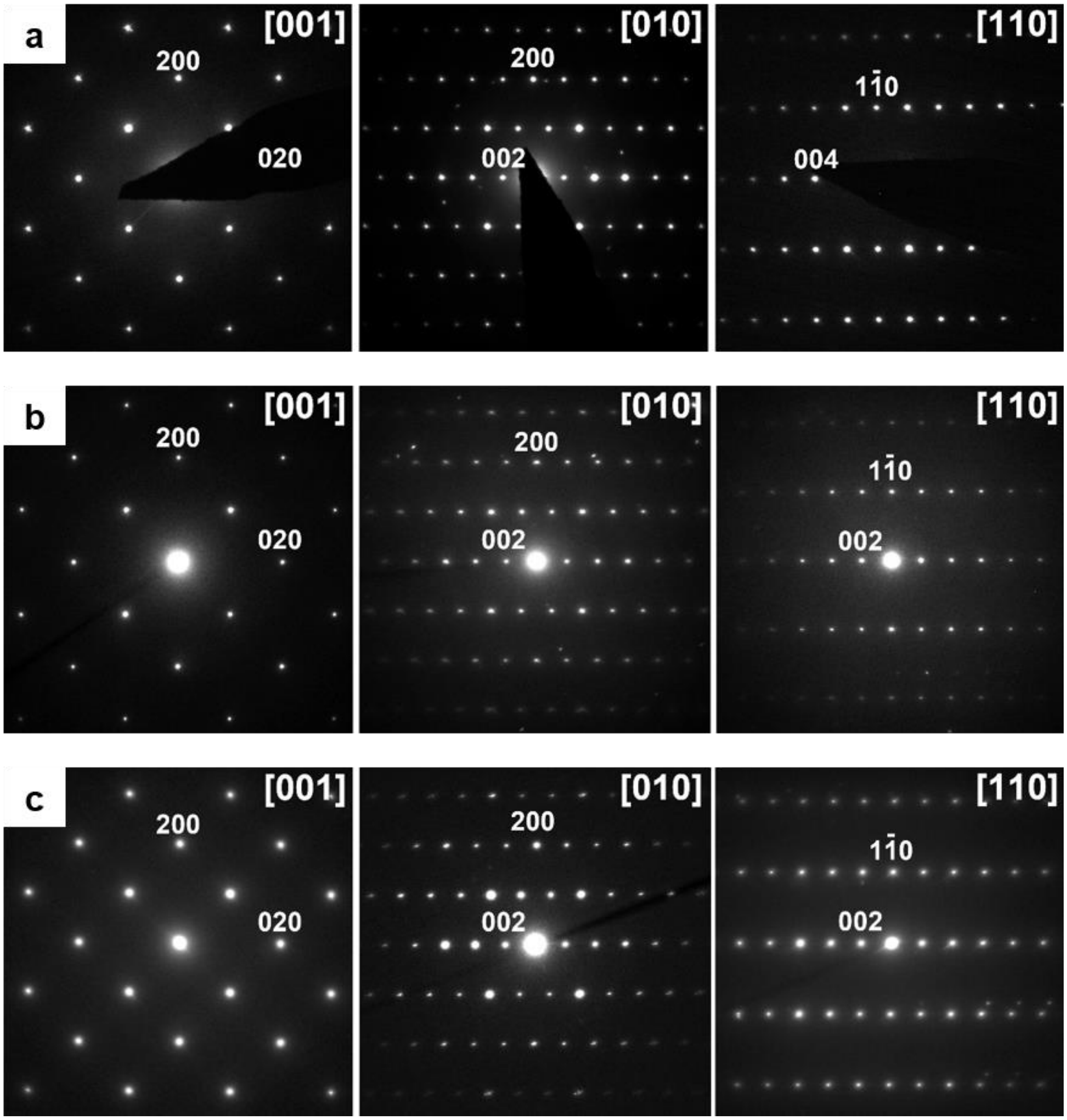

Supplementary Figure 1. Electron diffraction (ED) patterns of the LSNF series. **a** LSN. **b** LSNF45. **c** LSF. The ED patterns for LSNF30 are given in Figure 1 of the manuscript. The ED patterns are indexed in a body-centered tetragonal unit cell with the unit cell parameters listed in Supplementary Table1.

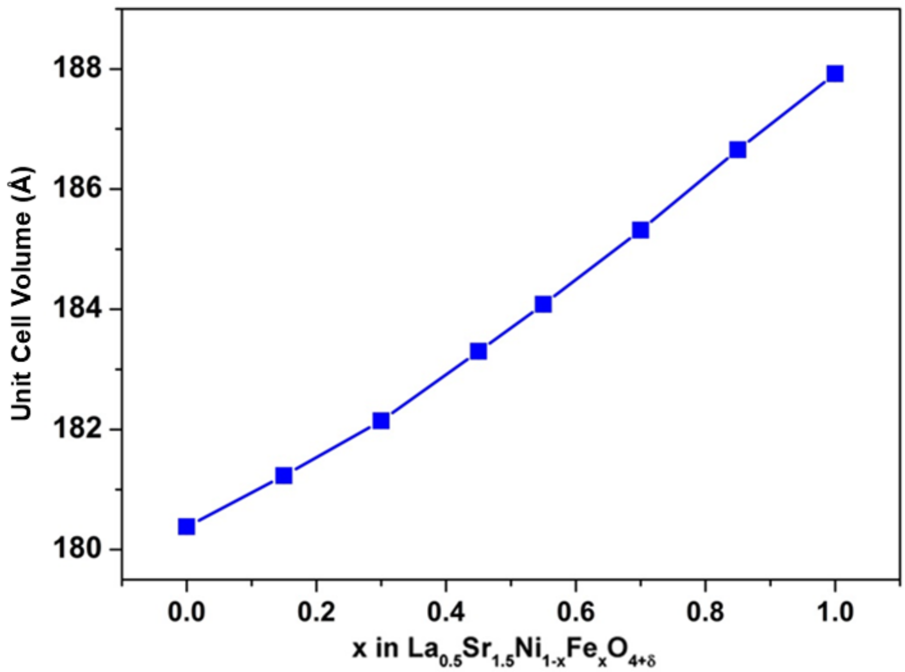

Supplementary Figure 2. Unit cell volume as a function of x in the $La_{0.5}Sr_{1.5}Ni_{1-x}Fe_xO_{4+\delta}$ solid solutions.

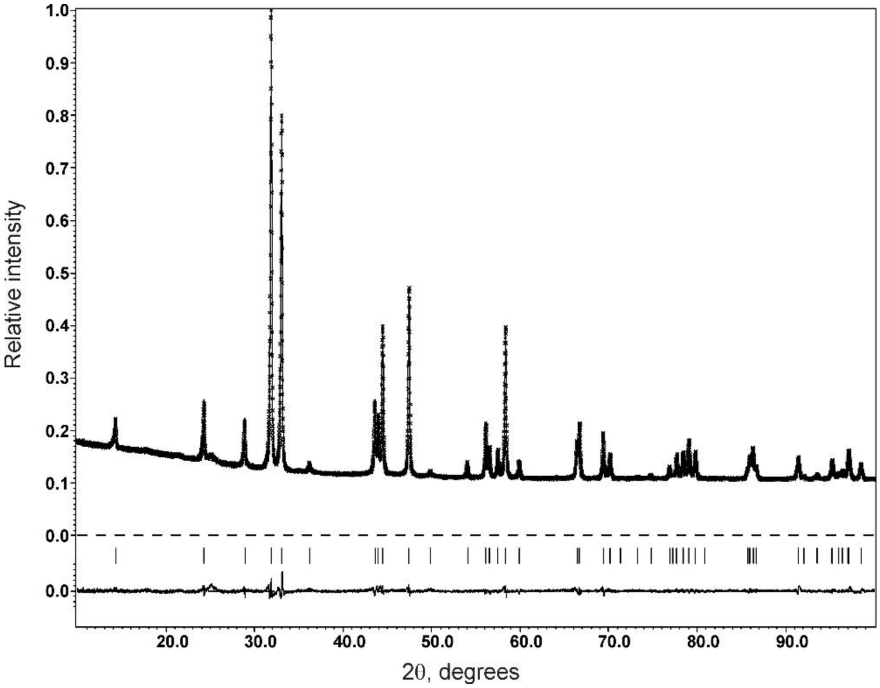

Supplementary Figure 3. Typical experimental, calculated and difference curves after the Rietveld refinement of the $La_{0.5}Sr_{1.5}Ni_{1-x}Fe_xO_{4+\delta}$ structures (exemplified for LSNF30). The bars mark the reflection positions of the *I*4/*mmm* structure.

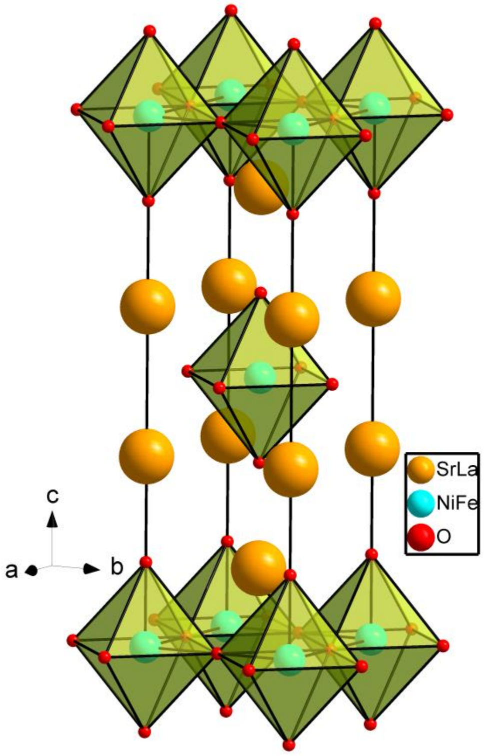

Supplementary Figure 4. The crystal structure of the *n*=1 Ruddlesden-Popper $La_{0.5}Sr_{1.5}Ni_{1-x}Fe_xO_{4+\delta}$ series.

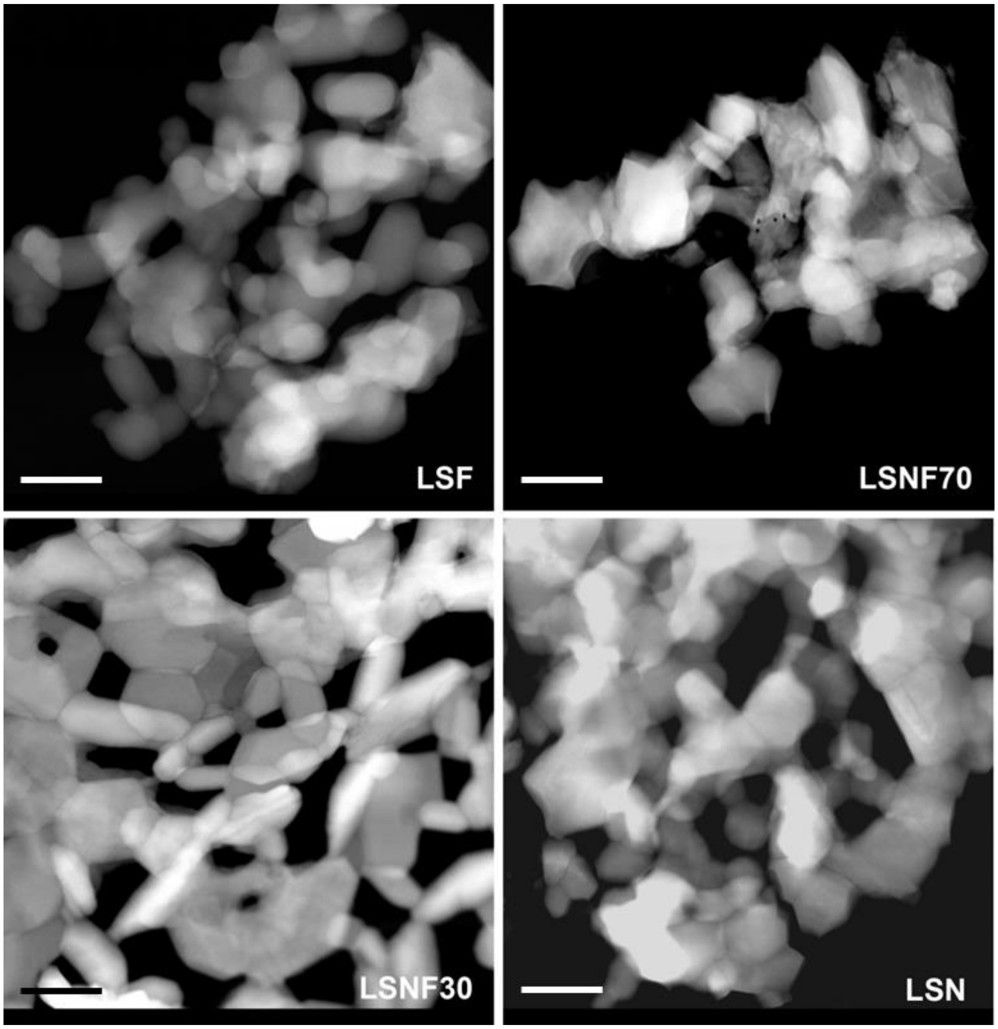

Supplementary Figure 5. HAADF-STEM images of $La_{0.5}Sr_{1.5}Ni_{1-x}Fe_xO_{4+\delta}$. Scale bars are 200 nm. The images demonstrate that the catalysts are comprised of similarly sized primary particles that form loosely sintered aggregates with well-developed porosity

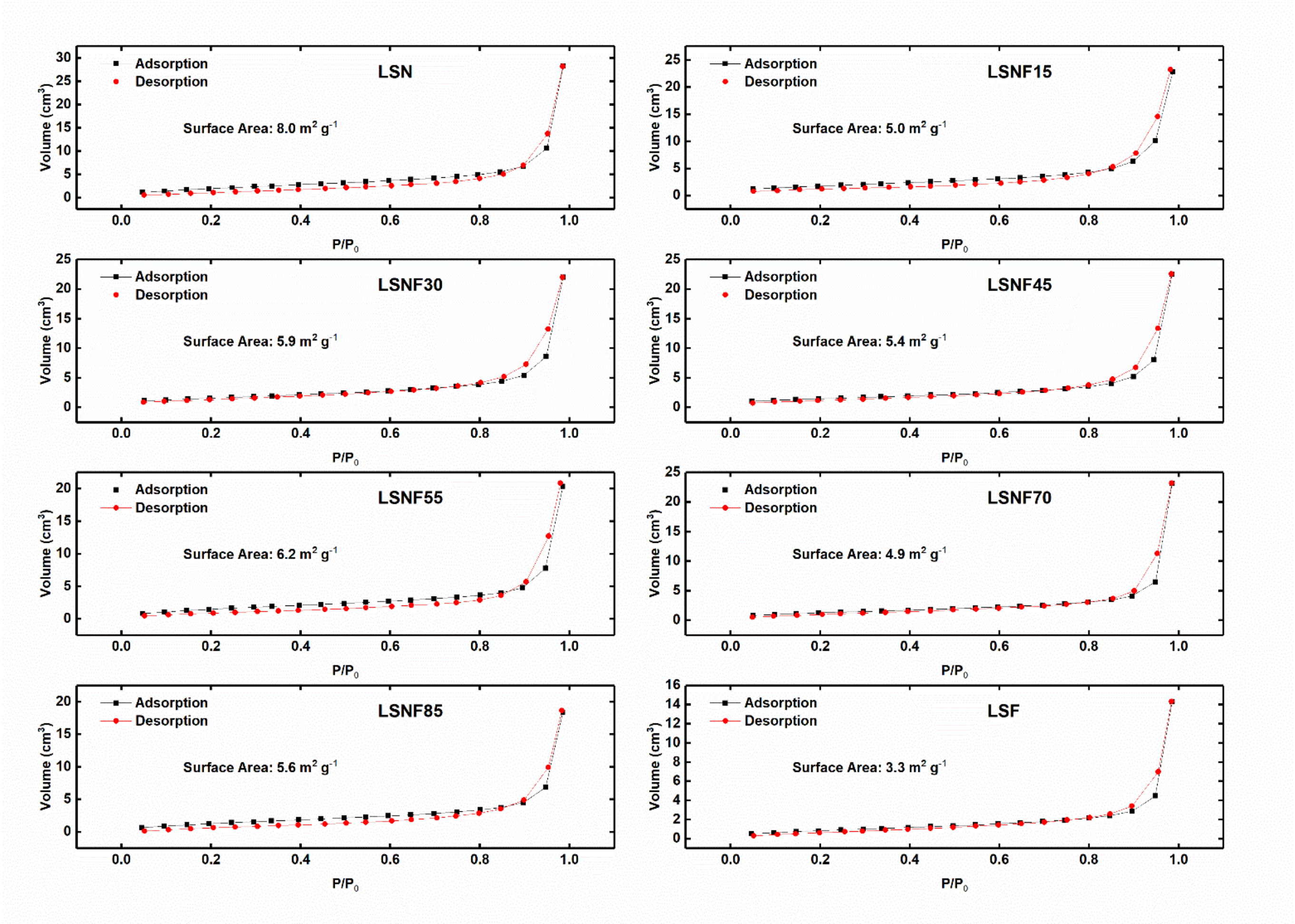

Supplementary Figure 6. Nitrogen sorption isotherms for BET surface area analysis of the $La_{0.5}Sr_{1.5}Ni_{1-x}Fe_xO_{4+\delta}$ series. Samples range in surface area from 3.3 to 8 m^2 g^{-1}. All samples underwent the same thermal treatments for mixed metal oxide precursor particle synthesis as well as crystallization and annealing.

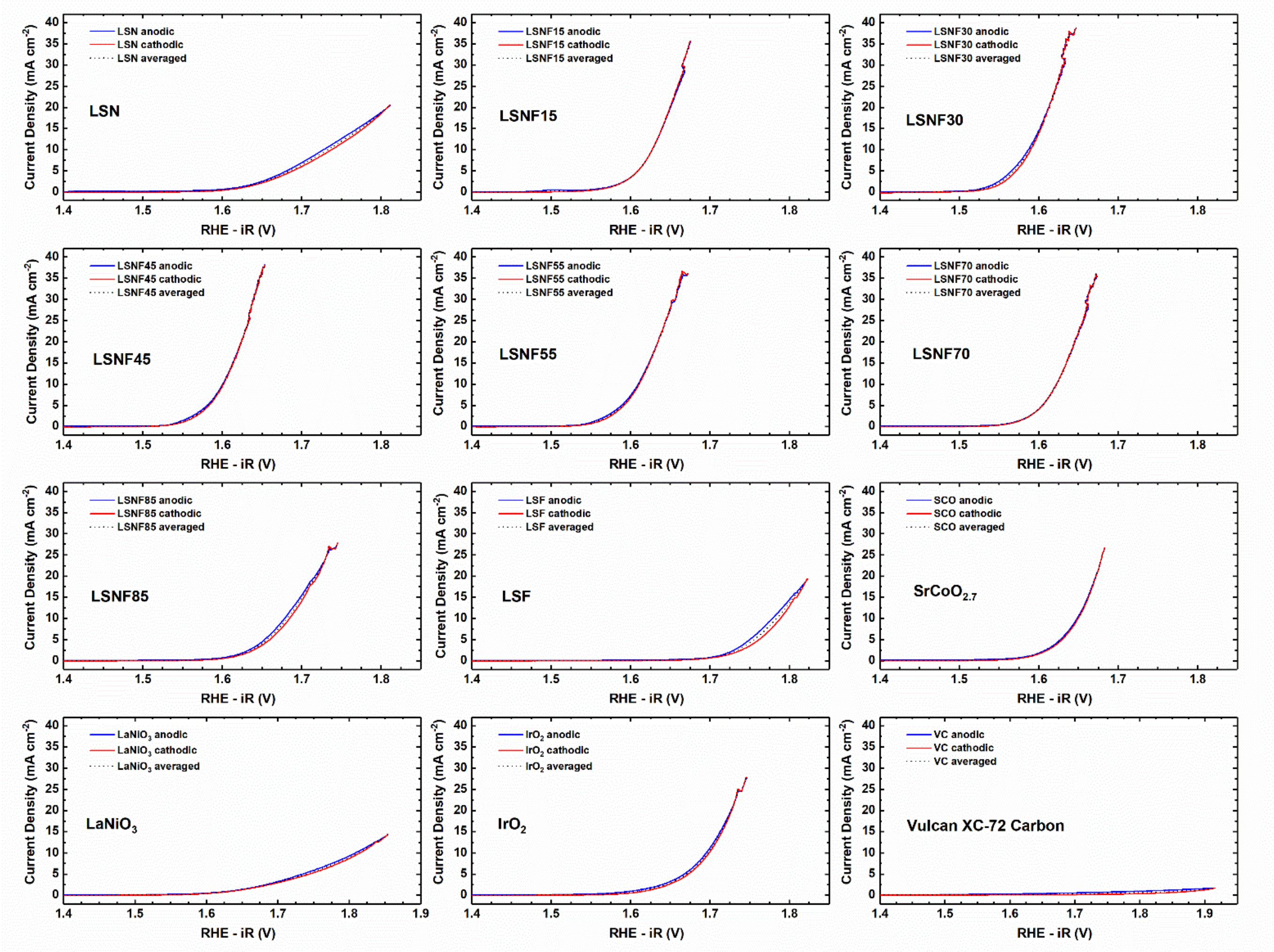

Supplementary Figure 7. Representative polarization curves taken in O_2 saturated 0.1 M KOH at 1600 rpm and 10 mV s^{-1}. Catalysts were first swept positive (anodic), then negative (cathodic), and the curves averaged before iR correction. Electrolyte resistance was measured to be 46 Ω. Total electrode loading is 51 μg_{total} cm^{-2}_{geo} with 30 wt% LSNF or SCO on XC72 Vulcan Carbon (VC), yielding 15.3 μg_{oxide} cm^{-2}_{geo}. IrO_2 was tested at 20 wt% on VC (10.2 μg_{oxide} cm^{-2}_{geo}) and neat VC was tested at 35.7 μg_{carbon} cm^{-2}_{geo} (7 µg carbon, corresponding to the contribution to measured OER current for 30 wt% LSNF on VC). $LaNiO_3$ and $SrCoO_{2.7}$ are the same materials as previously reported,[22, 33] except supported on VC to only probe the effects of catalyst composition.

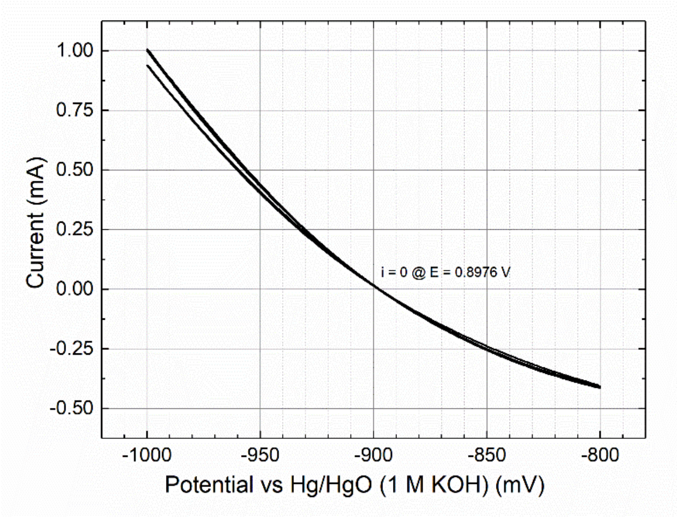

Supplementary Figure 8. Reversible hydrogen electrode (RHE) calibration. A Pt working electrode was cycled at 1 mV s^{-1} in H_2 saturated 0.1 M KOH using a Pt or Au counter electrode to standardize the Hg/HgO (1 M KOH) reference electrode against thermodynamic H_2 evolution and oxidation. The Hg/HgO conversion to RHE was determined by the average potential at which 0 current was measured, -0.8976 V vs Hg/HgO.

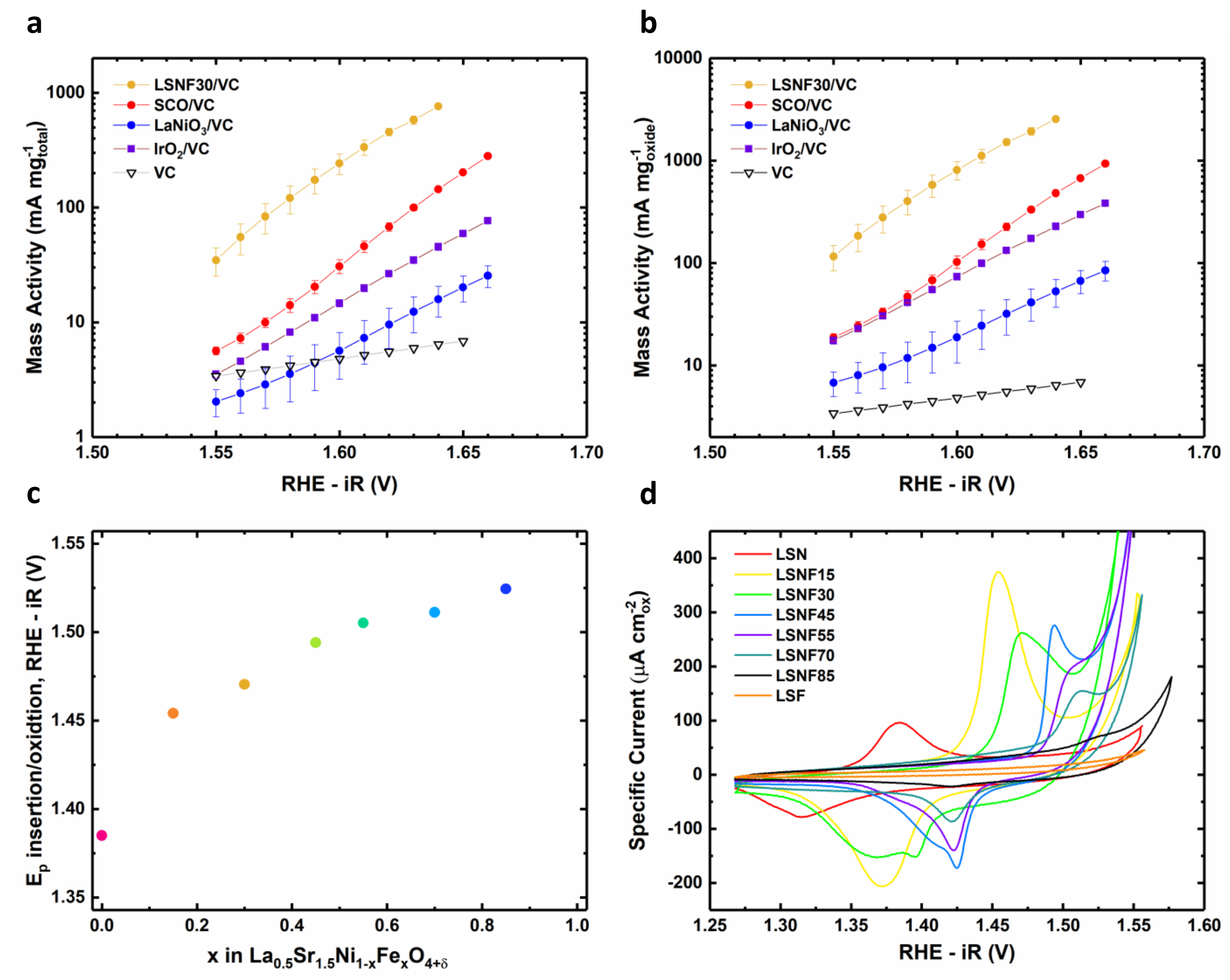

Supplementary Figure 9. OER Tafel plots for LSNF30 and other leading metal oxide catalysts and $Ni^{2+/3+}$ redox CVs and peak potentials for the LSNF series. **a** OER Tafel plot normalized on a on a total mass and **b** on a catalyst mass basis. All data in a-b taken in oxygen saturated 0.1 M KOH at 10 mV s^{-1} and 1600 rpm. LSNF30, SCO and $LaNiO_3$ are 30 wt% on VC, IrO_2 is 20 wt%, all corresponding to 51 μg_{total} $cm^{-2}{}_{geo}$. VC is XC72 Vulcan Carbon. Pure VC is tested at 35.7 μg cm^{-2}, corresponding to the 70 wt% carbon used to support LSNF30, SCO and $LaNiO_3$, and the mass activity should be read as mA $mg^{-1}{}_{carbon}$. **c** Shifting of the $Ni^{2+/3+}$ redox peak potentials as a function of Fe substitution. All peak potentials were taken from stable CVs at 10 mV s^{-1} in oxygen saturated 0.1 M KOH. For LSNF55 and LSNF85, there was no local maximum at 10 mV/s and the peak potential was selected after the OER background was subtracted out, shown in Supplementary Figure 16. **d** Stable intercalation CVs plotted in specific current density. All CVs are at 10 mV s^{-1} in O_2-saturated 0.1 M KOH.

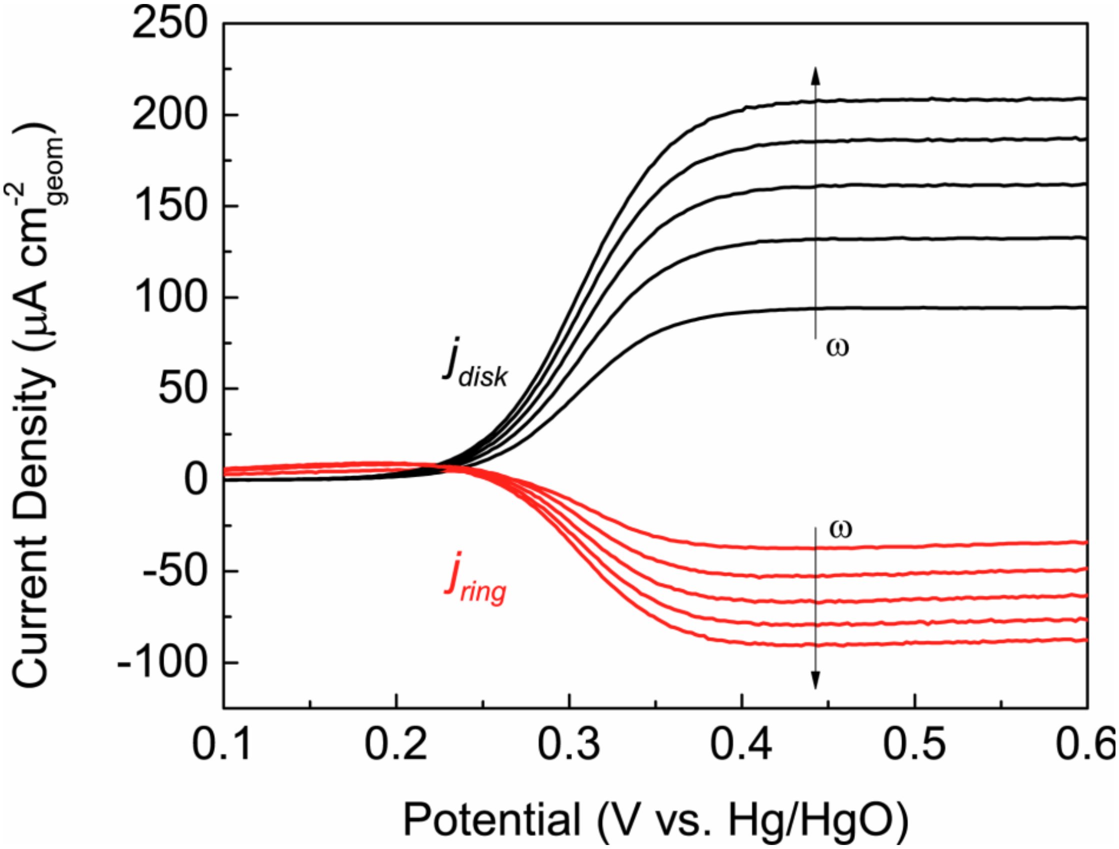

Supplementary Figure 10. Calibration of RRDE with Ferrocene-methanol. Linear sweep voltammetry was performed using a rotating ring-disk electrode (RRDE) with a glassy carbon disk and a Pt ring in 0.3 mM ferrocene-methanol and 0.1M KCl. The disk potential was scanned from 0.1 to 0.6 V vs. Hg/HgO (1M KOH) while the ring was held at 0.1 V vs. Hg/HgO (1M KOH). Rotation rates of ω = 400, 800, 1200, 1600, and 2000 rpm were used. The collection efficiency was measured as N=0.37.

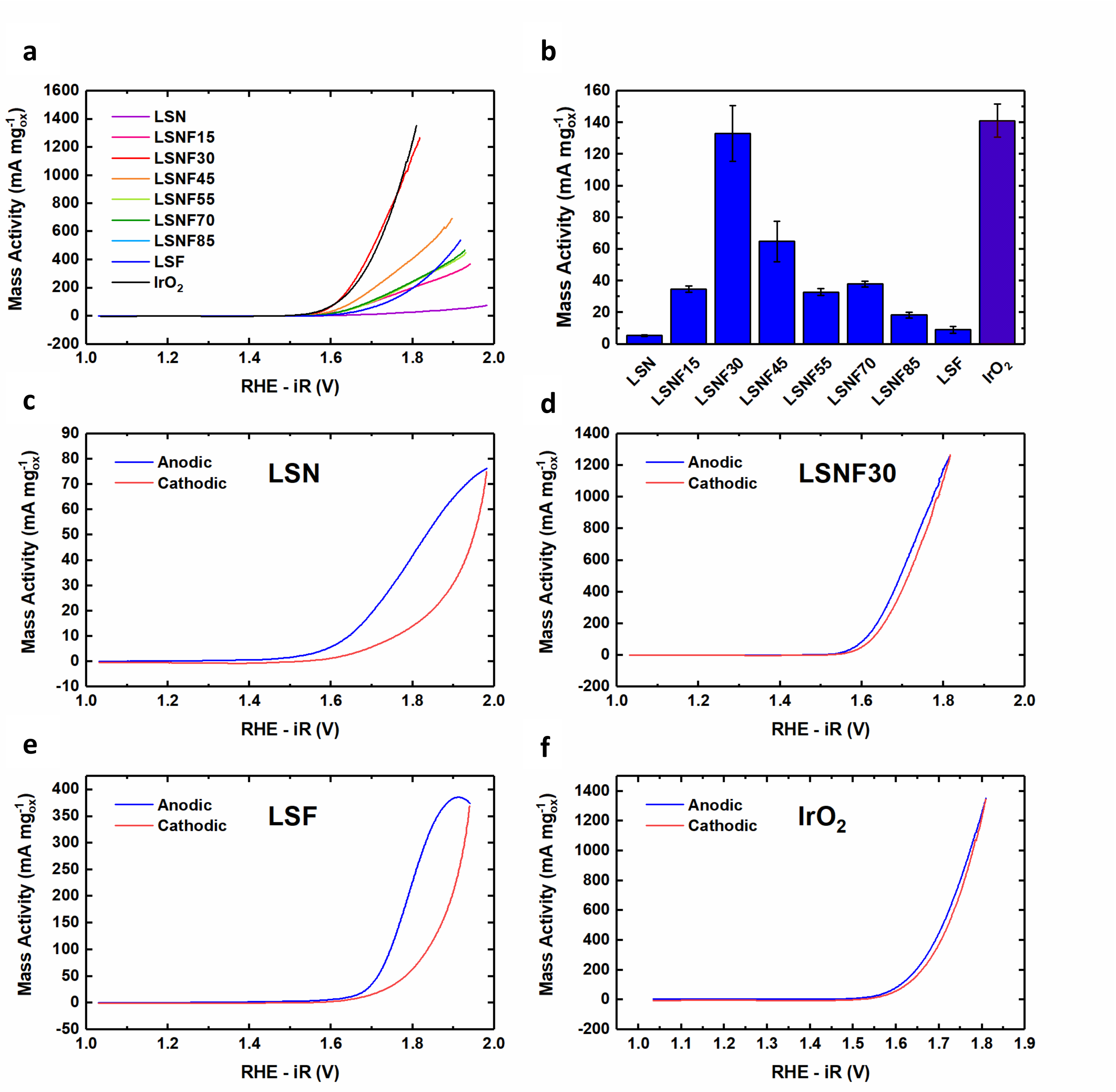

Supplementary Figure 11. Oxygen evolution results and catalytic activities for the unsupported LSNF series. **a** Activities measured from averaged anodic and cathodic scans at 1.63 V vs. RHE after iR correction in O_2 saturated 0.1 M KOH at 10 mV s^{-1} and 1600 rpm with a mass loading of 15.3 ug_{ox} cm^{-2}. **b** Bar graph of activities measured at 1.63 V. All measurements were performed in triplicate and averaged and error bars are the calculated standard deviations of these averages. **c-f** Representative CVs for LSN, LSNF30, LSF, and IrO_2 before averaging are shown.

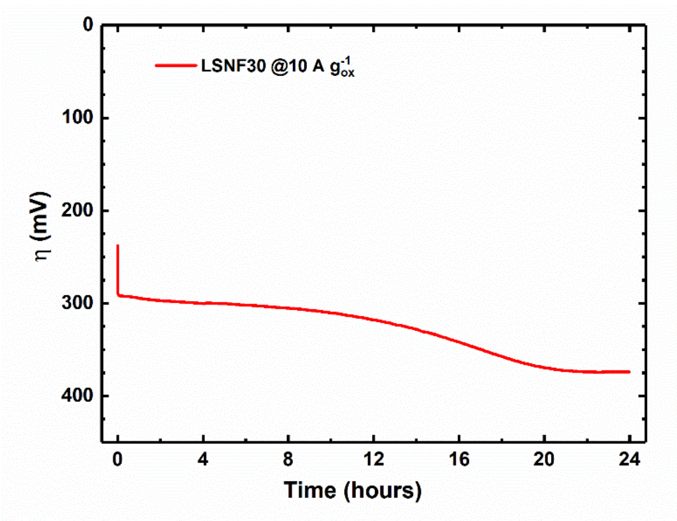

Supplementary Figure 12. Galvanostatic stability test of LSNF30 at 10 A/g_{oxide} in O_2 saturated 0.1 M KOH at 1600 rpm. LSNF30 was supported at 30 wt% on XC72 VC and dropcast onto a 5 mm GCE for a total mass loading of 51 μg cm^{-2}_{geo}.

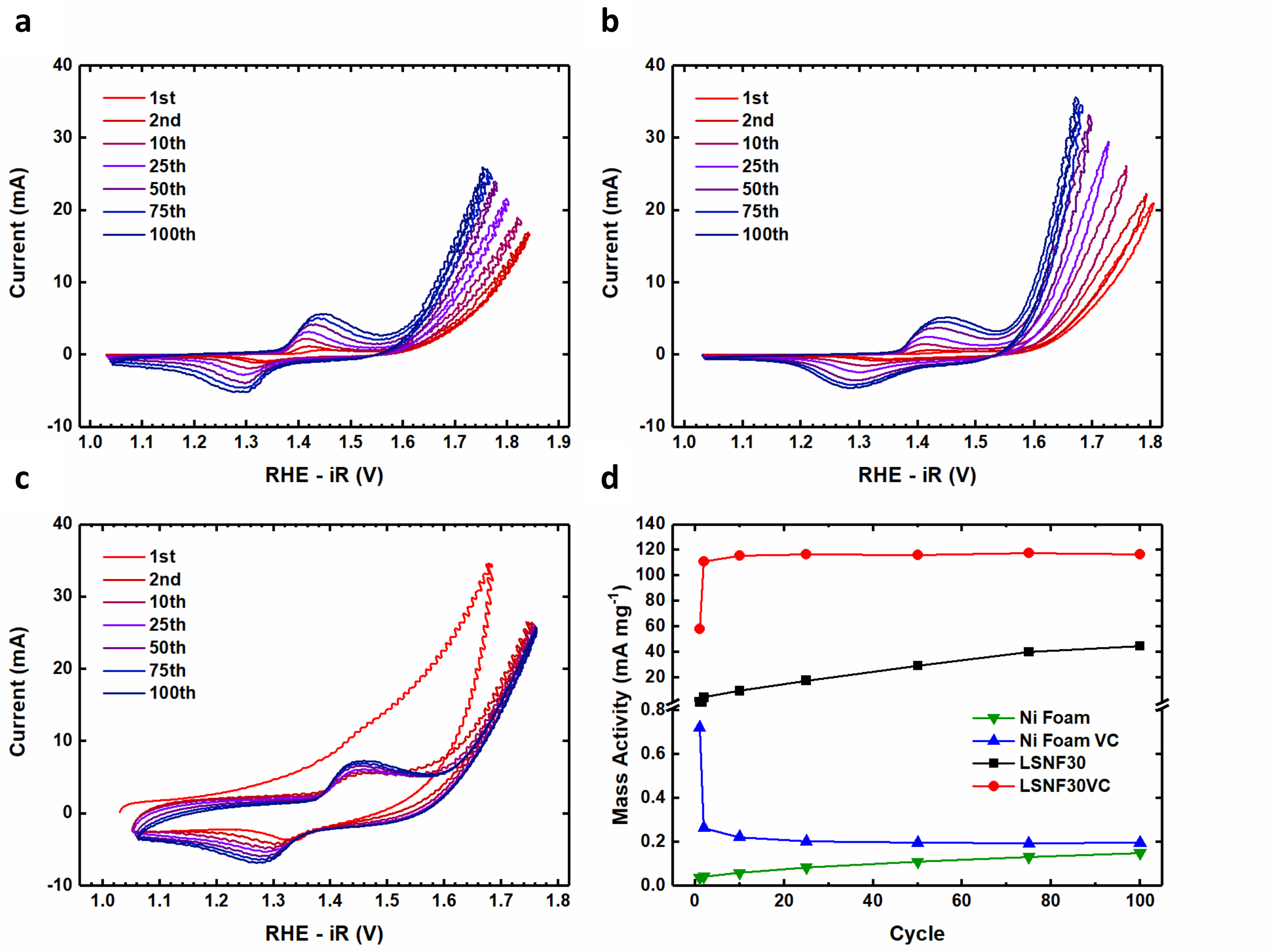

Supplementary Figure 13. Cyclic Voltammetry Stability tests of LSNF30 dropcast on Ni Foam electrodes and cycled in O_2-saturated 0.1 M KOH at 10 mV s^{-1}. **a** 100 cycles of a bare Ni foam electrode. **b** 100 cycles of a Ni foam electrode drop cast with 0.3 mg LSNF30. **c** 100 cycles of a Ni foam electrode drop cast with 0.7 mg of VC. **d** Mass activities for all four types of prepared Ni foam electrodes. Mass activities for the electrodes without LSNF30 were calculated by dividing the current by the mass of the Ni foam electrode. Mass activities for the LSNF were calculated by subtracting the current at 1.63 V for a Ni foam electrode with or without carbon but without catalyst from the current measured from the catalyst on Ni Foam with or without the VC support and then dividing by the mass of catalyst used.

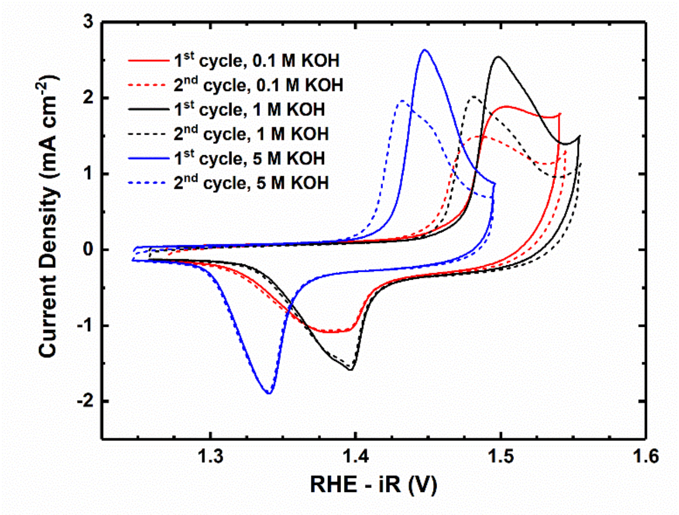

Supplementary Figure 14. First and second CVs of LSNF30 in various concentrations of KOH demonstrating the dependence of oxidation wave peak potential (E_p) on pH, consistent with OH^- intercalation. Shifts in peak potential between the first and second cycle indicate an initial amount of catalyst surface restructuring.

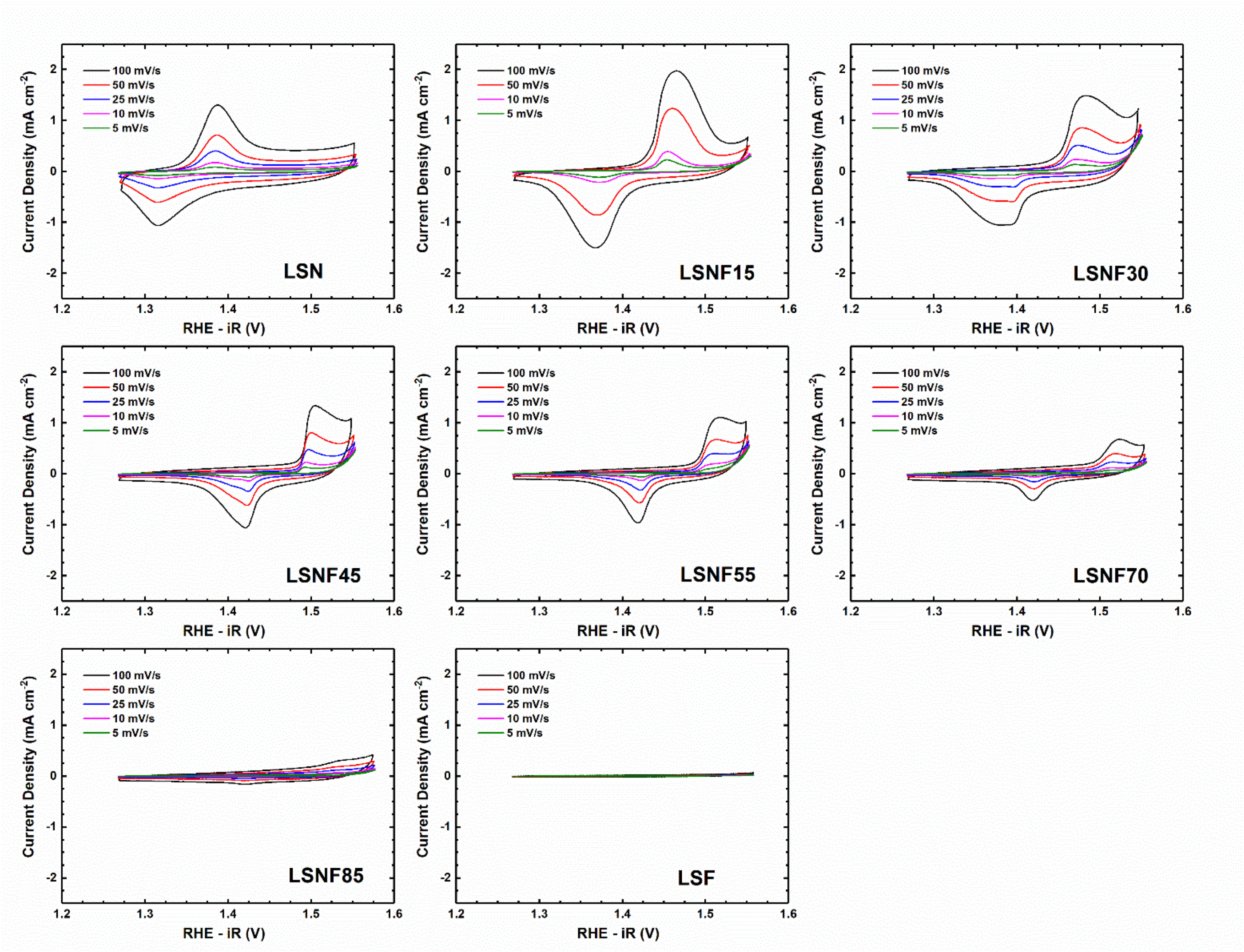

Supplementary Figure 15. Intercalation CVs of 30 wt% oxide on VC taken in O_2 saturated 0.1 M KOH after 3 - 4 cycles of preconditioning so that oxidation peak potentials do not change upon subsequent cycling. CVs were taken on pristine electrodes immediately following preconditioning and recorded for varying scan rates, descending from 100 mV s^{-1} to 5 mV s^{-1}, one electrode per material.

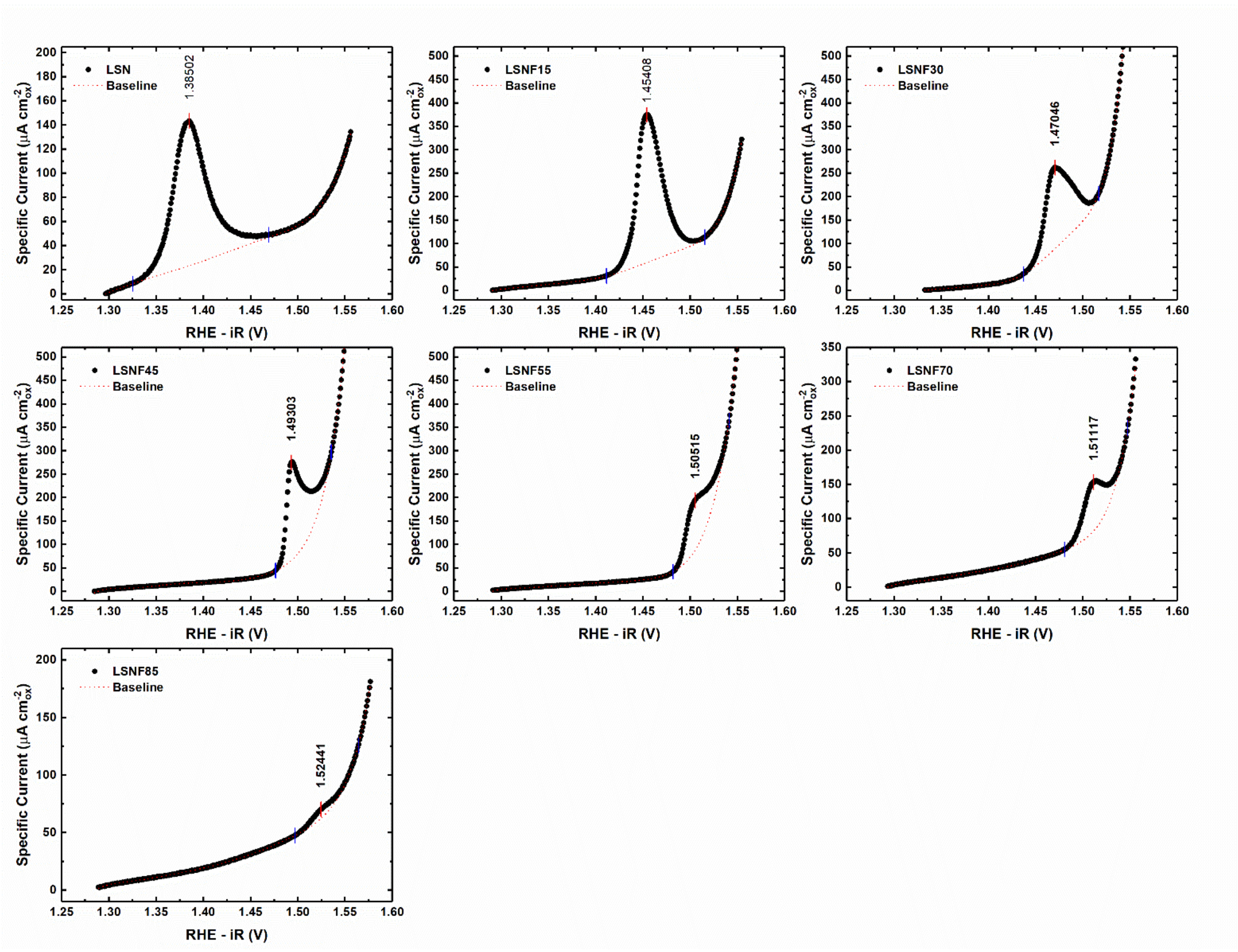

Supplementary Figure 16. Fitted intercalation CVs for $La_{0.5}Sr_{1.5}Ni_{1-x}Fe_xO_4$. The fitted baseline was subtracted prior to integration of peak areas. Currents were converted into specific activity to account for surface area differences between samples with varying Fe contents. All CVs are in O_2 saturated 0.1 M KOH and were taken at 10 mV s^{-1}. These are the same CVs from Supplementary Figure 15, meaning they were collected from a pristine electrode that was conditioned and cycled at 100, 50, and 25 mV s^{-1}. Consult the methods section for more information. Numbers are the computed peak maxima after baseline OER subtraction, used for determination of E_P in Supplementary Figure 9c for LSNF55 and LSNF85, in which no local maxima is observed prior to baseline subtraction.

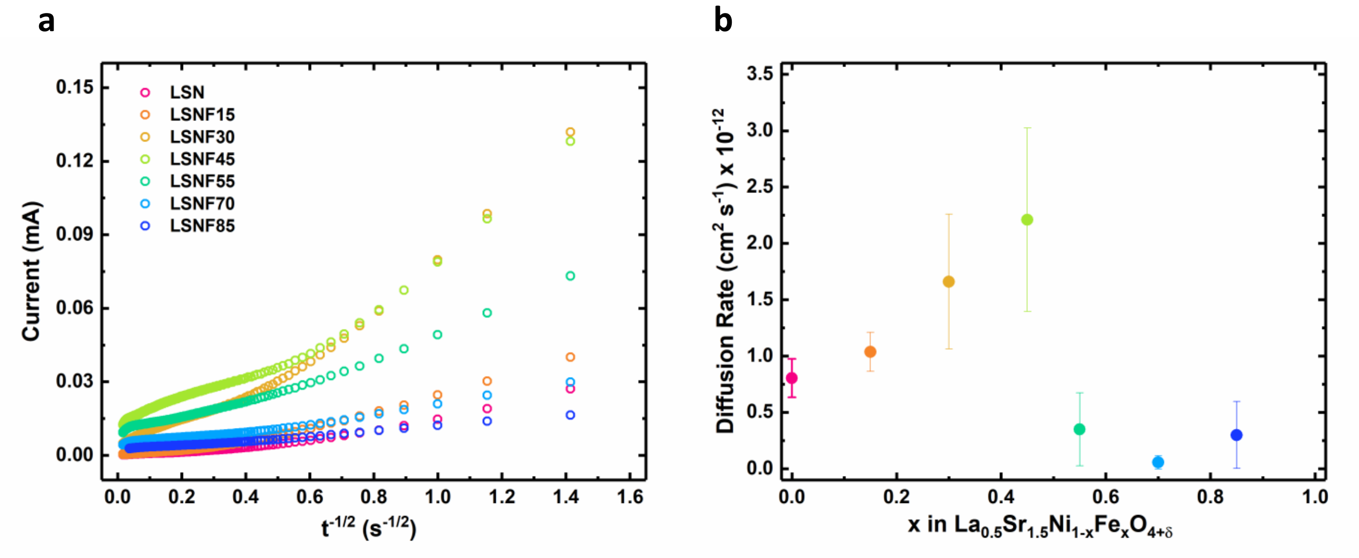

Supplementary Figure 17. Electrochemical diffusion rate data for the Sr-nickelate system taken in O_2 saturated 0.1 M KOH. **a** Calculated oxygen diffusion rates for LSNF catalysts. **b** Chronoamperometry data used for the calculation of diffusion rates in **a**. A linear regression was used on the linear, fast timescale portion of the current decay, and using the mathematical model referenced in the Methods section of the main text the diffusion rates were determined. Particle size was estimated from BET data and confirmed by the STEM images in Supplementary Figure 5. The shape factor (λ) was assumed to be 2 for all calculations. All materials are 85 wt% oxide on VC which were dropcast onto 5 mm glassy carbon electrodes (GCE) for a total loading of 51 μg cm^{-2}_{geo}.

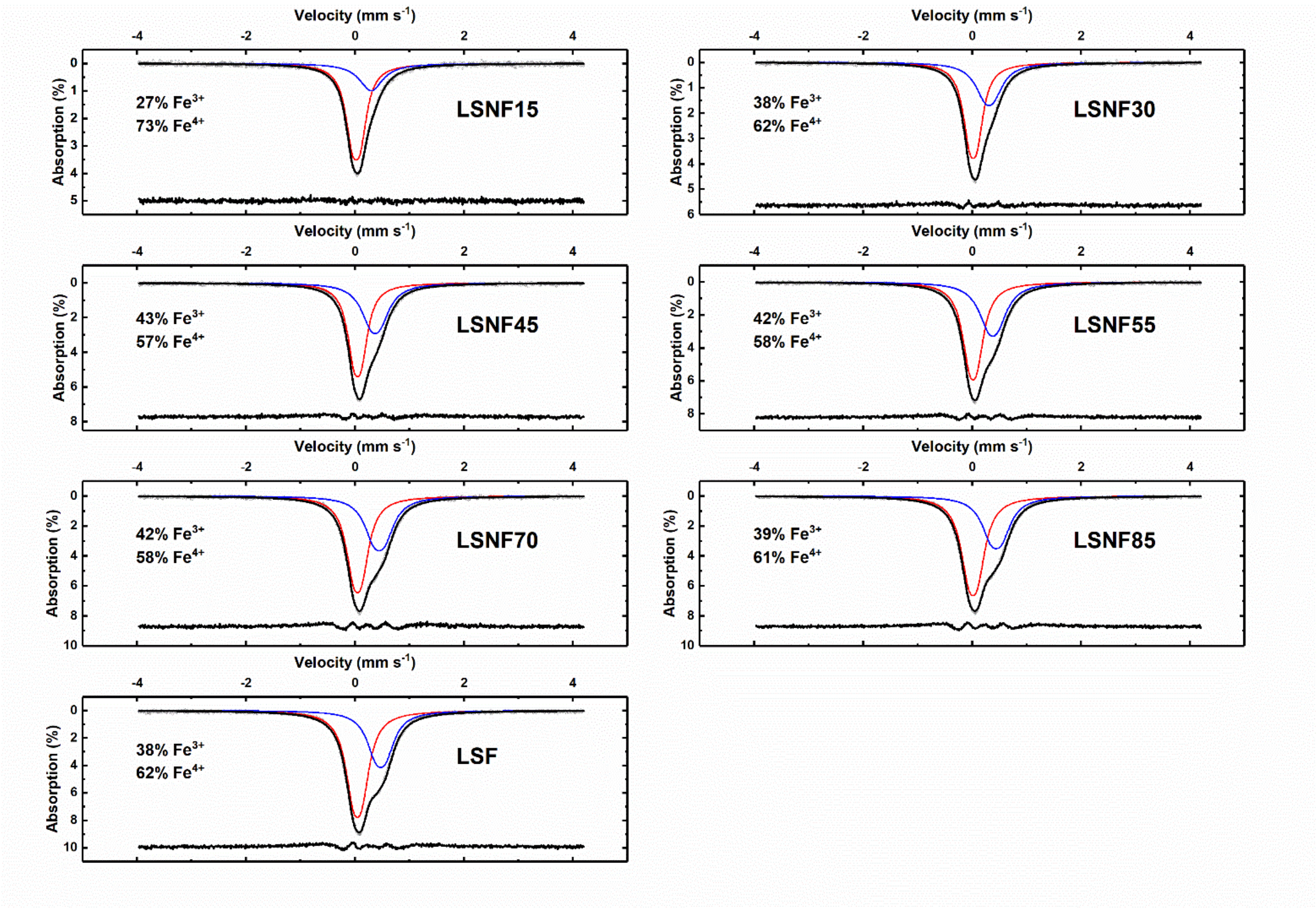

Supplementary Figure 18. Room temperature Mössbauer spectroscopy for the Sr-nickelate system. Fitted curves for Fe^{4+} depicted in red while fitted curves for Fe^{3+} are shown in blue. The spectra, measured at room temperature, were deconvoluted into two overlapped paramagnetic doublets. According to their chemical isomer shifts (ISs), the doublets with smaller ISs of ~0.02 - 0.04 mm/s correspond to Fe^{4+} cations, while ones with larger ISs of ~0.32 - 0.45 mm/s correspond to Fe^{3+} cations.[20] It was previously shown that values of quadrupole splitting (QSs) in the $Sr_{2-x}La_xFeO_{4\pm\delta}$ based solid solutions are highly dependent on La and O contents.[21] The observed values of hyperfine parameters, viz. ISs and QSs, for the Sr- nickelate system investigated are consistent with the parameters reported for the related $Sr_{2-x}La_xFeO_{4\pm\delta}$ compounds with high oxygen content.[22,23] It is to note that some of the samples were additionally measured at low temperature of 78K. The 78K Mossbauer spectra obtained were more difficult to analyze to resolve the Fe3+ and Fe4+ subspectra because of their higher broadening and overlapping, but no significant difference in the Fe3+/Fe4+ ratios at 78K and 298K were observed. For the sake of clarity, only the room temperature data are reported.

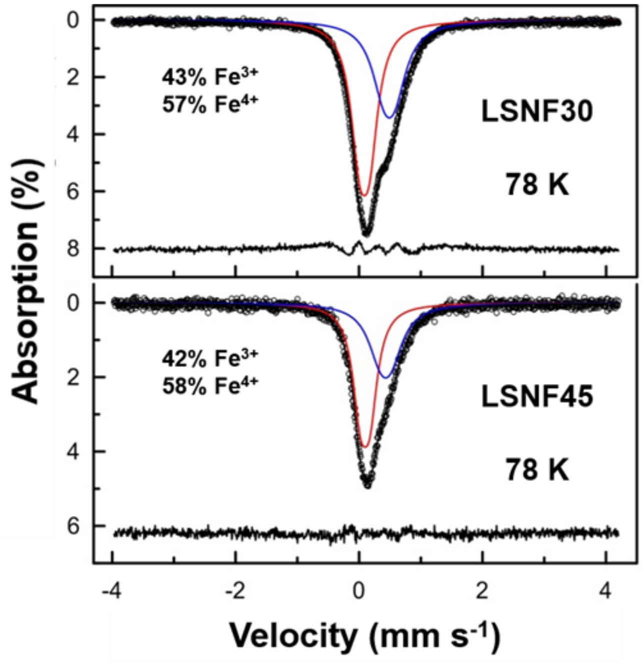

Supplementary Figure 19. Mössbauer spectroscopy taken at 78 K for LSNF30 and LSNF45, the two most active compositions for the Sr-nickelate system. Fitted curves for Fe^{4+} depicted in red while fitted curves for Fe^{3+} are shown in blue. The spectra were deconvoluted into two overlapped paramagnetic doublets in the same manner described in Supplementary Figure 16. The Mossbauer spectra obtained at 78K were more difficult to analyze to resolve the Fe3+ and Fe4+ subspectra because of their higher broadening and overlapping, but LSNF30 and LSNF45 were able to be analyzed and displayed very similar Fe3+/Fe4+ ratios at 78K and 298K and follow the trend observed for measurements taken across the entire series at room temperature.

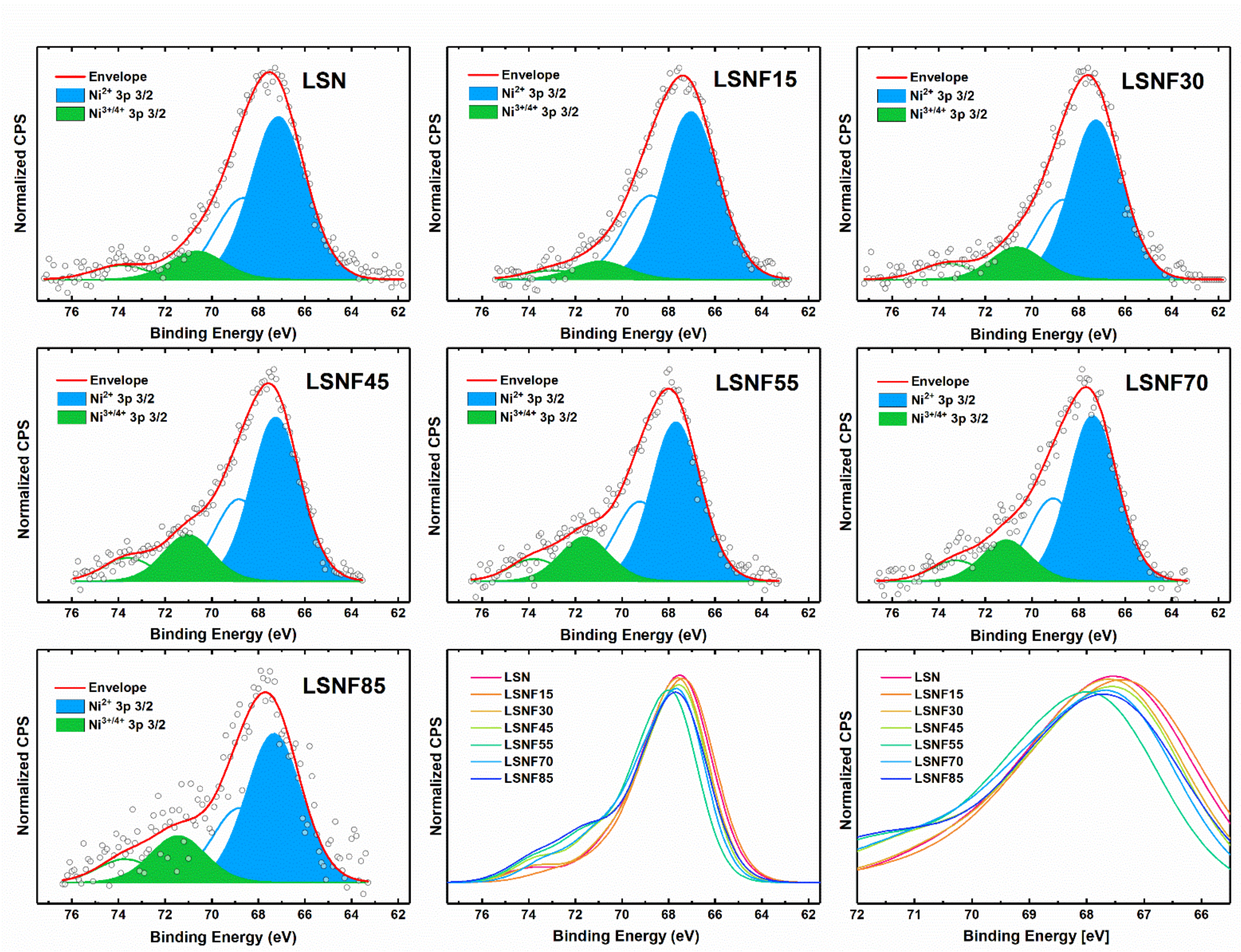

Supplementary Figure 20. XPS deconvolution of the Ni 3p spectra for $La_{0.5}Sr_{1.5}Ni_{1-x}Fe_xO_{4+\delta}$, performed by adaptation of the methods developed by Burriel et. al for $La_{2-x}Sr_xNiO_4$.[24] The Ni 3*p* spectrum was decomposed into 4 distinct components; Ni^{2+} and $Ni^{3+/4+}$ in both the $3p_{3/2}$ and $3p_{1/2}$ portions of the Ni doublet. Deconvolution was achieved by fitting error minimization on the 30% Gaussian/Lorentzian components using the Marquardt and Simplex methods within CasaXPS. To ensure self-consistency all components used the same FWHM and components assigned to Ni $3p_{1/2}$ were constricted to have exactly half the area of their counterparts in the Ni $3p_{3/2}$. Binding energy constraints were the same used by Burriel et. al, and the maximum allowed FWHM was increased to account for the multiple chemical states encompassed by the $Ni^{3+/4+}$ components.

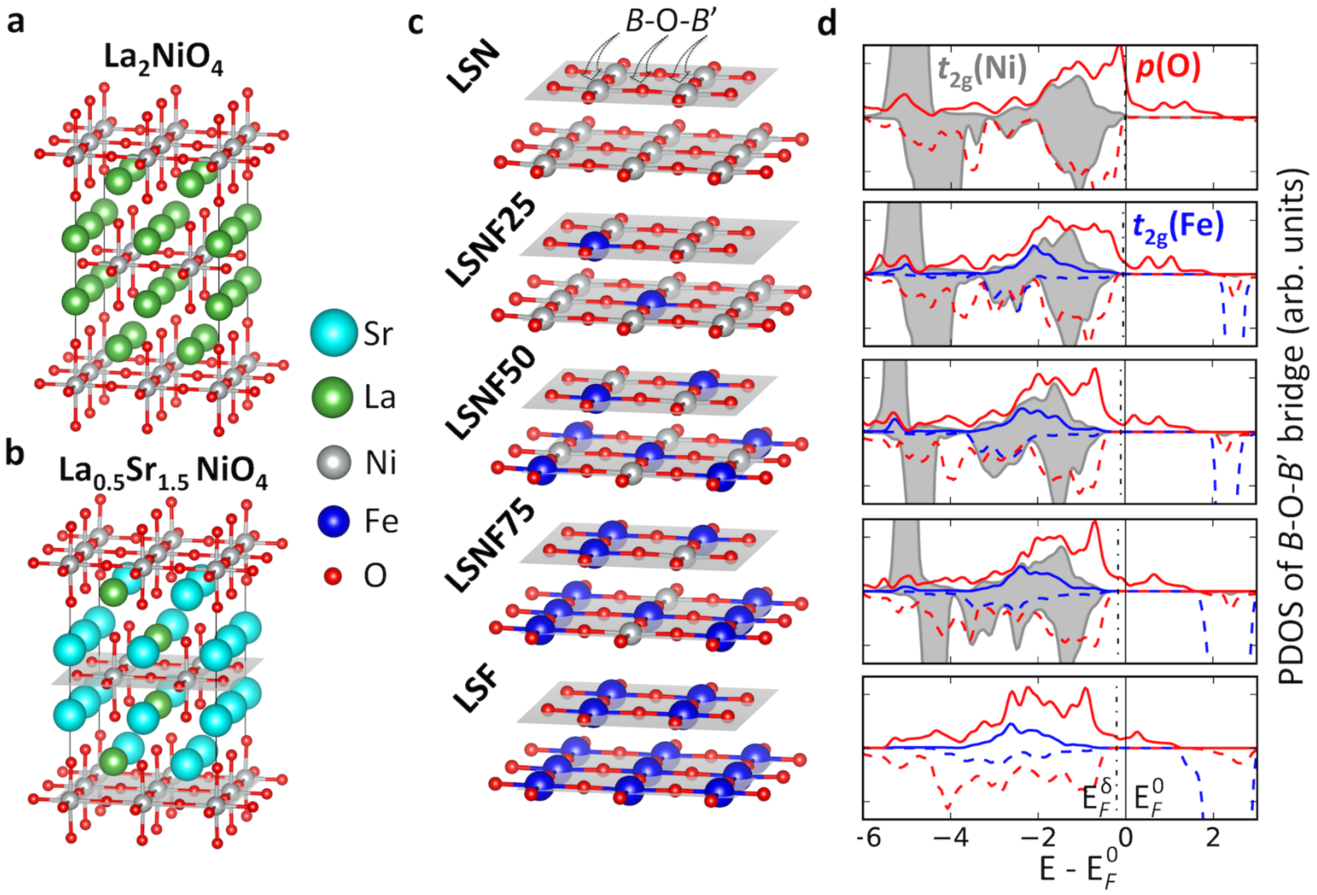

Supplementary Figure 21. DFT modeling of atomic and electronic structures of bulk LSNF. **a** The optimized atomic structure of 2x2x1 La_2NiO_4, as the initial structure for Sr substitution and relaxation. **b** and **c** follow the same as those in Figure 5. **d** The projected density of states (PDOS) of t_{2g} (Ni and Fe) and 2*p* (O) with respect to Fermi level for the *B*-O-*B'* bridges; t_{2g} is shown not to come across Fermi level.

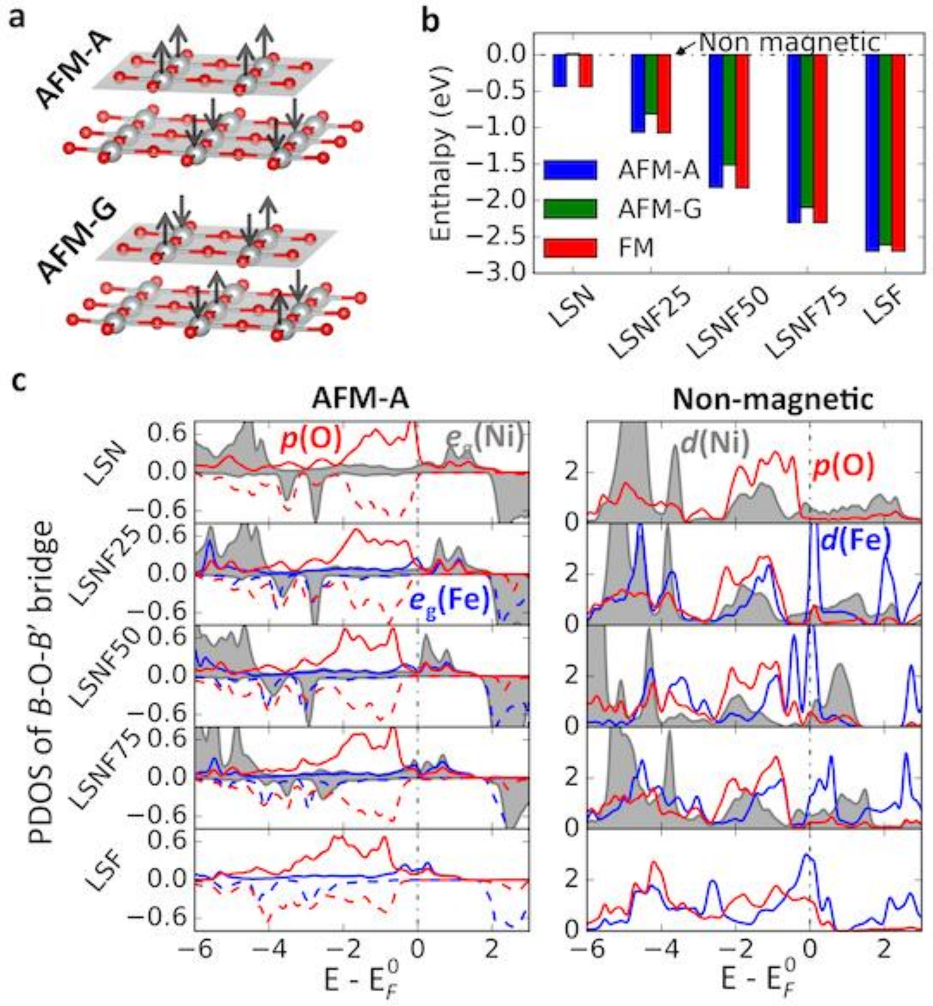

Supplementary Figure 22. Comparison of ferromagnetic, anti-ferromagnetic and non-magnetic structures from DFT modeling. **a** The structures of A-type antiferromagnetic (AFM-A) and G-type antiferromagnetic (AFM-G) orderings. In AFM-A, spin directions only differ between neighborhood BO_2 layers, while in AFM-G, spin directions differ between neighborhood *B* atoms; AFM-C is identical to AFM-G in this atomic structure. **b** Comparison of energetics per unit $La_{0.5}Sr_{1.5}Ni_{1-x}Fe_xO_4$, relative to the corresponding non-magnetic energy in each composition. The energetic comparison clearly indicates FM and AFM-A as more stable configurations. **c** The projected density of states (PDOS) of the *B*-O-*B*' bridges for both AFM-A and non-magnetic structures. PDOS of AFM-A is shown to be similar to that of FM in Figure 5, while that of non-magnetic structure gives consistent trend of bands movement as described for Figure 5.

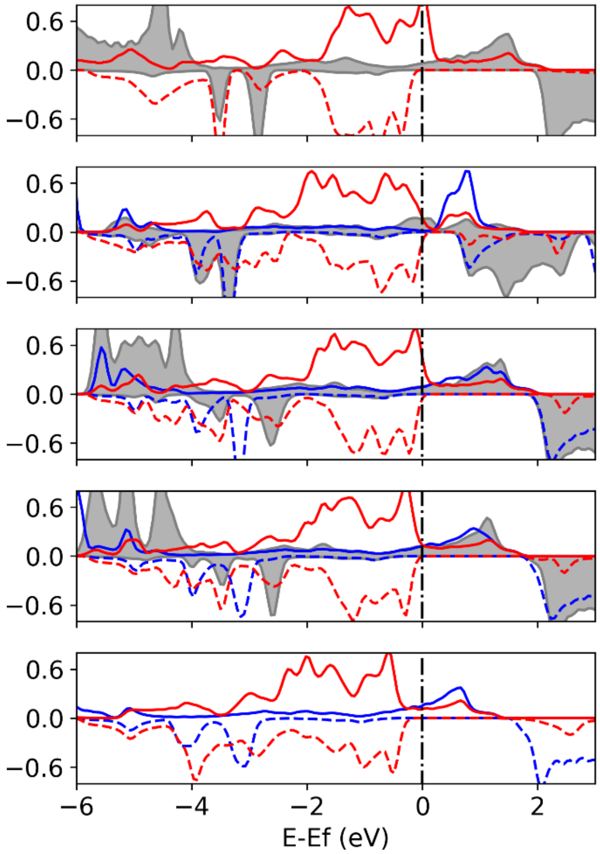

Supplementary Figure 23. DFT modeling of the electronic structures of bulk SNF. The projected density of states (PDOS) of e_g (Ni and Fe) and 2*p* (O) with respect to Fermi level for the *B*-O-*B’* bridges closely resemble that for the LSNF series presented in Figure 5 of the main text with only a slight downward shift of the Fermi level with increased B-site oxidation states due to full Sr substitution.

Supplementary Table 1. The results of Rietveld refinement for $La_{0.5}Sr_{1.5}Ni_{1-x}Fe_xO_{4+\delta}$. The refinement was performed in the *I*4/*mmm* space group with the atomic positions La,Sr 4*e* (0, 0, *z*), Ni,Fe 2*a* (0,0,0), O1 4*c* (0, ½, 0) and O2 4*e* (0, 0, *z*).

Sample	*a* [Å]	*c* [Å]	V [Å^3]	Refined composition	*z/c* (La,Sr)	*z/c* (O2)	*d*(M-O)$_{eq}$ [Å]	*d*(M-O)$_{ap}$ [Å]	R_F, R_P, R_{wP}
LSN	3.82437(2)	12.3331(1)	180.382(2)	$La_{0.518(6)}Sr_{1.482(6)}NiO_4$	0.35979(3)	0.1608(2)	1.91219(2)	1.984(2)	0.013, 0.010, 0.014
LSNF15	3.82806(2)	12.3673(1)	181.231(2)	$La_{0.496(6)}Sr_{1.504(6)}Ni_{0.85}Fe_{0.15}O_4$	0.35951(3)	0.1609(2)	1.91403(2)	1.990(2)	0.015, 0.011, 0.016
LSNF30	3.83130(2)	12.4085(1)	182.143(3)	$La_{0.518(6)}Sr_{1.482(6)}Ni_{0.70}Fe_{0.30}O_4$	0.35924(3)	0.1619(2)	1.91565(2)	2.009(2)	0.020, 0.012, 0.018
LSNF45	3.83506(2)	12.4629(1)	183.300(1)	$La_{0.534(6)}Sr_{1.466(6)}Ni_{0.55}Fe_{0.45}O_4$	0.35863(3)	0.1619(2)	1.91753(2)	2.017(3)	0.018, 0.012, 0.017
LSNF55	3.83724(2)	12.5018(2)	184.082(3)	$La_{0.500(6)}Sr_{1.500(6)}Ni_{0.45}Fe_{0.55}O_4$	0.35821(4)	0.1605(2)	1.91862(2)	2.007(3)	0.022, 0.013, 0.017
LSNF70	3.84143(2)	12.5581(2)	185.315(3)	$La_{0.524(8)}Sr_{1.476(8)}Ni_{0.30}Fe_{0.70}O_4$	0.35773(4)	0.1613(2)	1.92071(2)	2.026(3)	0.021, 0.012, 0.017
LSNF85	3.84533(2)	12.6232(2)	186.654(3)	$La_{0.524(6)}Sr_{1.476(6)}Ni_{0.15}Fe_{0.85}O_4$	0.35748(4)	0.1615(2)	1.92267(2)	2.039(3)	0.019, 0.013, 0.017
LSF	3.84789(3)	12.6919(2)	187.920(3)	$La_{0.536(6)}Sr_{1.464(6)}FeO_4$	0.35704(4)	0.1626(2)	1.92394(2)	2.064(3)	0.017, 0.013, 0.018

Supplementary Table 2. Comparison of LSNF30 with other promising OER catalysts and accompanying notes.

Catalyst	Support[A]	Surface Area [m^2/g]	Catalyst Loading [mg_{ox}/cm^2_{geo}]	η @ 10 mA/cm^2_{geo} [mV]	Specific Activity @ η = 400 mV [mA/cm^2_{ox}]	Mass Activity @ η = 400 mV [mA/mg_{ox}]	OER Tafel slope [mV/dec]	Testing Conditions	Ref.
$La_{0.5}Sr_{1.5}Ni_{0.7}Fe_{0.3}O_{4.04}$	VC	5.9	0.015	360	32.7	1930	44	0.1 M KOH, O_2 sat, 10 mV/s at 1600 rpm; iR corrected	This work
$SrCoO_{2.7}$	VC	3.6	0.015	419	9.4	332	67	0.1 M KOH, O_2 sat, 10 mV/s at 1600 rpm; iR corrected	This work
$LaNiO_3$	VC	11	0.015	582	0.4	41	99	0.1 M KOH, O_2 sat, 10 mV/s at 1600 rpm; iR corrected	This work
IrO_2	VC	14.5	0.010	468	1.2	175	88	0.1 M KOH, O_2 sat, 10 mV/s at 1600 rpm; iR corrected	This work
$SrSc_{0.025}Nb_{0.025}Co_{0.95}O_{3-\delta}$	C65	0.1	0.360	411	58	58	55-60	0.1 M KOH, O_2 sat, 10 mV/s at 1600 rpm; iR corrected	25
$BaNiO_3$ / $Ba_2Ni_2O_5$	VC	0.3	0.295	421	4.2 (9.1)[B]	13 (27)	--	0.1 M KOH, O_2 sat, 10 mV/s at 1600 rpm; iR corrected	26
$Pr_{0.5}Ba_{0.5}CoO_{3-\delta}$	AB	0.35	0.250	>381[C]	9.1[D]	31[E]	60	0.1 M KOH, O_2 sat, 10 mV/s at 1600 rpm; iR corrected	27
$CaCu_3Fe_4O_{12}$	AB	0.45	0.250	378	15.5	70	59 (44)[B]	0.1 M KOH, O_2 sat, 10 mV/s at 1600 rpm; iR corrected	28
$Ca_{1.75}Pr_{0.25}MnO_{4\pm\delta}$	AB	1.8	0.400	>471[C]	14.5E-3	0.26	86	0.1 M KOH, O_2 sat, 10 mV/s at 1600 rpm; iR corrected	29
$La_{1.2}Ca_{0.8}NiO_4$	C65	10.8	0.397	373	18.8	52.2	42	0.1 M KOH, O_2 sat, 10 mV/s at 1600[F] rpm; iR corrected	30
$La_{0.7}(Ba_{0.5}Sr_{0.5})_{0.3}Co_{0.8}Fe_{0.2}O_{3\pm\delta}$	KB	21.3	0.639	375	0.12	25	97	0.1 M KOH, O_2 sat, 10 mV/s at 1600 rpm; iR corrected	31
Ni-Fe LDH (Ni:Fe 45:55)	--	27	0.051[G]	311	~1.1[H]	~295[I]	37	0.1 M KOH, O_2 sat, 2 mV/s at 1600 rpm; iR corrected	32

[A] Supports: **VC** - Cabot XC-72 Vulcan Carbon; **C65** - TIMCAL Super C65; **AB** - Acetylene Black; **KB** - Ketjenblack EC-600JD

[B] Results in ( ) are after cycling

[C] Current density did not reach 10 mA/cm^2_{geo}, overpotential listed is maximum tested

[D] Potential only reported to 1.61 V, LSNF30's corresponding value is 19 mA/cm^2_{ox}

[E] Potential only reported to 1.61 V, LSNF30's corresponding value is 1100 mA/mg_{ox}

[F] Electrodes rotation rate not reported

[G] Mass was determined only by metal weight, neglecting O(H) contributions. Calculated mass and specific activities will be larger than truly measured.

[H] Current density only reported to ~1.55 V, LSNF30's corresponding value is 2.0 mA/cm^2_{ox}

[I] Current density only reported to ~1.55 V, LSNF30's corresponding value is 115 mA/mg_{ox}

Supplementary Table 3. Summary of the physical, chemical and electrochemical properties of the LSNF series.

**Sample**	**Surface Area [m^2/g]**	**Ni^{AVG+}**	**Oxygen Excess [δ]**	**E_P $Ni^{2+/3+}$ oxidation [V]**	**Electrochemical Oxygen Diffusion Rate [cm^2/s]**	**Specific Activity @ 1.63 V [mA/cm^2_{ox}]**	**Mass Activity @ 1.63 V [mA/mg_{ox}]**
LSN	8.0	3.54 ± 0.03	0.018 ± 0.013	1.385	8.0E-13 ± 1.7E-13	1.1 ± 0.0	87.7 ± 1.2
LSNF15	6.8	3.57 ± 0.02	0.046 ± 0.007	1.454	1.0E-12 ± 1.7E-13	10.6 ± 0.8	717.8 ± 54.6
LSNF30	5.9	3.57 ± 0.03	0.042 ± 0.010	1.470	1.7E-12 ± 6.0E-13	32.7 ± 2.8	1930.8 ± 164.0
LSNF45	5.4	3.68 ± 0.10	0.065 ± 0.027	1.494	2.2E-12 ± 8.2E-13	27.4 ± 0.8	1480.7 ± 45.7
LSNF55	6.2	3.80 ± 0.05	0.088 ± 0.011	1.505	3.5E-13 ± 3.2E-13	18.4 ± 0.6	1143.7 ± 40.2
LSNF70	4.9	3.82 ± 0.14	0.076 ± 0.022	1.511	5.6E-14 ± 6.0E-14	17.7 ± 1.7	868.4 ± 85.1
LSNF85	5.6	3.95 ± 0.48	0.081 ± 0.036	1.524	3.0E-13 ± 3.0E-13	2.3 ± 0.2	128.8 ± 12.3
LSF	3.3	--	0.143 ± 0.037	--	--	0.3 ± 0.1	10.4 ± 3.9

Surface area determined by multipoint BET measurements.
Average Ni oxidation state determined by a combination of iodometric titrations and deconvolution of room temperature Mössbauer spectroscopy.
Oxygen excess determined by iodometric titrations.
Peak potential for $Ni^{2+/3+}$ oxidation determined from CVs taken at 10 mV/s and after iR correction applied.
Oxygen diffusion rates determined electrochemically as outlined in the Methods section of the main text and the SI.

Supplementary Table 4. The 298K temperature ^{57}Fe Mossbauer hyperfine parameters for the Sr-nickelate system: IS, isomer shift relative to α-Fe at ambient temperature; ΔE_Q, apparent quadrupole shift; Γ, line width; I, relative spectral area.

Sample	Comp.	IS, mm/s ± 0.02	ΔE_Q, mm/s ± 0.02	I, % ± 2	Γ, mm/s ± 0.02	Comments
LSNF15	D11	0.03	0.15	73	0.30	Fe4+
	D12	0.31	0.14	27	0.47	Fe3+
LSNF30	D21	0.03	0.14	62	0.30	Fe4+
	D22	0.32	0.16	38	0.45	Fe3+
LSNF45	D31	0.03	0.14	57	0.30	Fe4+
	D32	0.35	0.17	43	0.45	Fe3+
LSNF55	D41	0.03	0.14	58	0.34	Fe4+
	D42	0.39	0.17	42	0.47	Fe3+
LSNF70	D51	0.03	0.15	58	0.35	Fe4+
	D52	0.43	0.20	42	0.44	Fe3+
LSNF85	D61	0.03	0.16	61	0.36	Fe4+
	D62	0.45	0.19	39	0.44	Fe3+
LSF	D71	0.03	0.16	62	0.36	Fe4+
	D72	0.45	0.18	38	0.41	Fe3+

Supplementary Table 5. The 78K temperature ^{57}Fe Mossbauer hyperfine parameters for LSNF30 and LSNF45 of the Sr-nickelate system: IS, isomer shift relative to α-Fe at ambient temperature; ΔE_Q, apparent quadrupole shift; Γ, line width; I, relative spectral area.

Sample	Comp.	IS, mm/s ± 0.02	ΔE_Q, mm/s ± 0.02	I, % ± 2	Γ, mm/s ± 0.02	Comments
LSNF30	D13	0.11	0.15	57	0.31	Fe4+
	D14	0.44	0.17	43	0.49	Fe3+
LSNF45	D13	0.10	0.16	58	0.34	Fe4+
	D14	0.50	0.18	42	0.48	Fe3+

Supplementary Table 6. Computed oxygen vacancy formation for modeled LSNF compositions at Fe-O-Ni bridge, relative to that for pseudo-cubic $LaNiO_3$. $LaNiO_3$ is computed to have more negative O_{vac} formation energy than $La_{0.5}Sr_{0.5}CoO_{3-\delta}$.

**Sample**	ΔE_{V_O} (eV)
LSN	-0.46
LSNF25	-0.28
LSNF50	-0.04
LSNF75	0.28
LSF	0.75

Supplementary Note 1

Concerning the persistent oxygen hyperstoichiometry and high oxidation states measured for LSNF.

There are conflicting reports in the literature surrounding the average Ni oxidation state and oxygen hyperstoichiometry of $La_{2-x}Sr_xNiO_{4+\delta}$ and until the current study data were not available for $La_{0.5}Sr_{1.5}Ni_{1-x}Fe_xO_{4+\delta}$. The results in Supplementary Table 3 indicate that Ni and Fe in Sr-nickelate are highly oxidized and that oxygen hyperstoichiometry increases as more Fe is substituted for Ni. However, others have observed increasing oxygen hyperstoichiometry at room temperature with increasing Fe content in La-rich $(La,Sr)_2Ni_xFe_{1-x}O_{4+\delta}$.[1,2] Medarde and Rodriguez-Carvajal found that ordered oxygen vacancies formed in $La_{2-x}Sr_xNiO_{4-\delta}$ when $x > 0.13$, while Aguadera and coworkers observed initial oxygen hyperstoichiometry when $x = 0.25$ in $La_{2-x}Sr_xNiO_{4+\delta}$, but find all samples to be oxygen deficient at $x \geq 0.5$, in contrast with our results.[3–5] Other reports of $La_{2-x}Sr_xNiO_{4+\delta}$ demonstrate that Sr substitution increases the average oxidation state of Ni due to charge compensation, up to and including $La_{0.5}Sr_{1.5}NiO_{3.98}$ in which Ni has an average oxidation state of +3.46.[6–8] Synthetic methodology and calcination temperature were both demonstrated to significantly affect the oxygen stoichiometry and crystal structure of $La_2NiO_{4+\delta}$,[9] and a similar conclusion was reached by Inprasit in rationalizing their oxygen hyperstoichiometry in $La_{1.2}Sr_{0.8}NiO_{4.2}$.[7] For $La_{2-x}Sr_xNiO_{4+\delta}$ synthesized using solid-state methods and crystallized at higher temperatures, lower Ni oxidation states and oxygen substoichiometry were observed.[10] Thus, we rationalize that the good molecular intermixing promoted by the polymerizable complex synthesis, the pure oxygen calcination environment, and our relatively low crystallization temperature of 950° C and subsequent 6 hour oxygen anneal at 400° C contribute to the high oxygen content and higher oxidation states listed in Supplementary Table 3, as has been seen for related materials elsewhere.[10,11] Finally, there is precedence for Fe substitution increasing the average oxidation state of a more electronegative B-site in a related n=1 RP system, $NdSrCo_{1-x}Fe_xO_{4+\delta}$.[12] Song et al found that increasing Fe substitution led to an increase in oxygen content and B-site oxidation state, measured by iodometry, which is logically consistent with our results.

Supplementary Note 2

Density Functional Theory (DFT) modeling details: determinations of effective Hubbard U_{eff}, atomic, magnetic and electronic structures, and oxygen hyper-stoichiometry effects.

Due to DFT self-interaction errors for strongly correlated materials,[13] the DFT+U method is employed to understand the electronic structures of LSNF. The values of U_{eff} of ~6.0eV (Ni) and ~5.0eV(Fe) are chosen based on previous work: In the case of Ni-containing perovskites, Gou et al. found that using U_{eff} (Ni) of ~6.0 eV gives a $LaNiO_3$ electronic structure closest to that obtained from hybrid functional methods and experimental spectroscopic data.[14] Lee et al. showed that this choice of U_{eff} also gives the correct $LaNiO_3$ formation enthalpy.[15] For Fe-contained perovskites, Shein et al. used a U_{eff} (Fe) of ~5.0eV to reproduce the experimental band gap for $LaFeO_3$, as well as the experimental magnetic structures and moments for both $LaFeO_3$ and $SrFeO_3$.[16] This choice of U_{eff} is also suggested by Ritzmann et al, particularly for those perovskites that exhibit some Fe(IV) character.[17,18] It is noteworthy that moderate adjustment of U_{eff} in a typical reported range may not significantly alter the density of states around Fermi level, as compared between several studies using U_{eff} (Ni) from 5.7 to 6.4eV[14,15] and U_{eff} (Fe) from 4.3 to 5.4eV.[15–17]

We model the LSNF compounds using 2x2x1 primitive unit cells, which enables the unit composition $La_{0.5}Sr_{1.5}NiO_{4+\delta}$ (LSN), $La_{0.5}Sr_{1.5}Ni_{0.75}Fe_{0.25}O_{4+\delta}$ (LSNF25), $La_{0.5}Sr_{1.5}Ni_{0.50}Fe_{0.50}O_{4+\delta}$ (LSNF50), $La_{0.5}Sr_{1.5}Ni_{0.25}Fe_{0.75}O_{4+\delta}$ (LSNF75), and $La_{0.5}Sr_{1.5}FeO_{4+\delta}$ (LSF). Starting from a 2x2x1 cell of *I*4/*mmm* La_2NiO_4 (LN, Figure S16a), we first search for the ground state ordering of Sr substitutions for stoichiometric LSN through structural screening and relaxations. For the LSN, we find that La/Sr prefers a uniform (random) distribution with one La in each of the 4 *A*O layers, due to the ionic nature of La/Sr anions. We then perform a second round of screening to determine the minimum energy distributions of Fe substitutions for each stoichiometric LSNF composition.

Magnetic orderings, including ferromagnetic (FM), antiferromagnetic (AFM, A, C and G types) and non-magnetic structures are all investigated across the whole LSNF series to ensure that the magnetic ordering that gives rise to the lowest free energy structure is used in the calculation of PDOS diagrams. In the FM state, the overall spin directions of each individual *B* cation are aligned with each other. In the A-type AFM structure, the spins are parallel within each BO_2 layer, but antiparallel between neighboring BO_2 layers, as illustrated in Supplementary Figure 22. In the G-type AFM state, the spins of neighboring *B* atoms are aligned antiparallel both in the plane of each BO_2 layer and between the BO_2 planes, forming a checkerboard pattern. In this crystal structure, C-type and G-type AFM are identical. Supplementary Figure 22b shows the computed energies of the

optimized LSNF cells with different magnetic states. As the figure shows, the FM and A-type AFM structures are very close in energy, while the G-type AFM and non-magnetic orderings are less stable across the whole LSNF series. This condition suggests that the large spatial separation leads to negligible dipole interactions between neighboring BO_2 layers. Thus, the in-plane charge density distribution is similar for the A-type AFM and FM cases, as shown by comparing the PDOS in Supplementary Figure 22c to that in Figure 5. Further investigation of the non-magnetic structure also reveals a similar trend in the *d*(B)-to-*p*(O) covalency from LSN to LSF, suggesting that characteristics of the PDOS for the FM state (shown in Figure 5 in the main text), are representative even above the Neel temperature.

We note that oxygen hyperstoichiometry effects either give rise to the emergence of bound states in conduction bands, a Fermi level shift, or a combination of the two.[19] In the former case, the overall electronic structure is subject to negligible change due to the dilute and localized nature of interstitial oxygen and bound states. In the latter case, the magnitude of the Fermi level shift can be roughly estimated via the rigid band model to accommodate unoccupied band formation, which can be determined by $E_F^\delta = E_F^0 - 2e^-\delta/\mathrm{DOS}(E_F^0)$, where $\mathrm{DOS}(E_F^0)$ is the total density of states at E_F^0 per formula unit of LSNF. As Figure 5c shows, the slightly adapted Fermi level, E_F^δ, does not qualitatively alter our discussions and conclusions. This situation is due to the rich band distribution of O-*p* holes and B-*d* states around the Fermi level.

Supplementary References

1. Benloucif, R., Nguyen, N., Greneche, J. M. & Raveau, B. La2−2xSr2xNi1−xFexO4−(x2) + δ: Magnetic and electron transport properties. *J. Phys. Chem. Solids* 50, 435–440 (1989).
2. Gilev, A. R., Kiselev, E. A. & Cherepanov, V. A. Homogeneity range, oxygen nonstoichiometry, thermal expansion and transport properties of La2−xSrxNi1−yFeyO4+δ. *RSC Adv.* 6, 72905–72917 (2016).
3. Medarde, M. & Rodríguez-Carvajal, J. Oxygen vacancy ordering in La2− x SrxNiO4 − δ (0 ≤ ≤ 0.5): the crystal structure and defects investigated by neutron diffraction. *Z. Phys. B* 102, 307–315 (1997).

4. Millburn, J. E., Green, M. A., Neumann, D. A. & Rosseinsky, M. J. Evolution of the Structure of the K2NiF4 Phases La2−xSrxNiO4+δ with Oxidation State: Octahedral Distortion and Phase Separation (0.2≤x≤1.0). *Journal of Solid State Chemistry* 145, 401–420 (1999).
5. Aguadero, A. *et al.* Effect of Sr content on the crystal structure and electrical properties of the system La2−xSrxNiO4+δ (0 ≤ x ≤ 1). *Dalton Trans.* 4377–4383 (2006). doi:10.1039/B606316K
6. Takeda, Y. *et al.* Crystal chemistry and physical properties of La2−xSrxNiO4 (0 ≤ x ≤ 1.6). *Mater. Res. Bull.* 25, 293–306 (1990).
7. Inprasit, T., Wongkasemjit, S., Skinner, S. J., Burriel, M. & Limthongkul, P. Effect of Sr substituted La2−xSrxNiO4+δ (x = 0, 0.2, 0.4, 0.6, and 0.8) on oxygen stoichiometry and oxygen transport properties. *RSC Adv.* 5, 2486–2492 (2014).
8. Manthiram, A., Tang, J. P. & Manivannan, V. Factors Influencing the Stabilization of Ni+ in Perovskite-Related Oxides. *Journal of Solid State Chemistry* 148, 499–507 (1999).
9. Fontaine, M.-L., Laberty-Robert, C., Ansart, F. & Tailhades, P. Elaboration and characterization of La2NiO4+δ powders and thin films via a modified sol–gel process. *Journal of Solid State Chemistry* 177, 1471–1479 (2004).
10. Sharma, I. B. & Singh, D. Solid state chemistry of Ruddlesden-Popper type complex oxides. *Bull. Mater. Sci.* 21, 363–374 (1998).
11. Mogni, L. *et al.* Synthesis, crystal chemistry and physical properties of the Ruddlesden–Popper phases Sr3Fe2−xNixO7−δ (0⩽x⩽1.0). *J. Solid State Chem.* 178, 1559–1568 (2005).
12. Song, K.-W. & Lee, K.-T. Characterization of NdSrCo1−xFexO4+δ (0≤x≤1.0) intergrowth oxide cathode materials for intermediate temperature solid oxide fuel cells. *Ceramics International* 37, 573–577 (2011).
13. Anisimov, V. I., Aryasetiawan, F. & Lichtenstein, A. I. First-principles calculations of the electronic structure and spectra of strongly correlated systems: the LDA + U method. *J. Phys.: Condens. Matter* 9, 767 (1997).

14. Gou, G., Grinberg, I., Rappe, A. M. & Rondinelli, J. M. Lattice normal modes and electronic properties of the correlated metal LaNiO${}_{3}$. *Phys. Rev. B* 84, 144101 (2011).

15. Lee, Y.-L., Kleis, J., Rossmeisl, J. & Morgan, D. Ab initio energetics of LaBO3 (001) (B = Mn, Fe, Co, and Ni) for solid oxide fuel cell cathodes. *Phys. Rev. B* 80, 224101 (2009).

16. Shein, I. R., Shein, K. I., Kozhevnikov, V. L. & Ivanovskii, A. L. Band structure and the magnetic and elastic properties of SrFeO3 and LaFeO3 perovskites. *Phys. Solid State* 47, 2082–2088 (2005).

17. Ritzmann, A. M., Muñoz-García, A. B., Pavone, M., Keith, J. A. & Carter, E. A. Ab Initio DFT+U Analysis of Oxygen Vacancy Formation and Migration in La1-xSrxFeO3-δ (x = 0, 0.25, 0.50). *Chem. Mater.* 25, 3011–3019 (2013).

18. Muñoz-García, A. B. *et al.* Unveiling Structure–Property Relationships in Sr2Fe1.5Mo0.5O6−δ, an Electrode Material for Symmetric Solid Oxide Fuel Cells. *J. Am. Chem. Soc.* 134, 6826–6833 (2012).

19. Malashevich, A. & Ismail-Beigi, S. First-principles study of oxygen-deficient ${\mathrm{LaNiO}}_{3}$ structures. *Phys. Rev. B* 92, 144102 (2015).

20. Menil, F. Systematic trends of the 57Fe Mössbauer isomer shifts in (FeOn) and (FeFn) polyhedra. Evidence of a new correlation between the isomer shift and the inductive effect of the competing bond T-X (→ Fe) (where X is O or F and T any element with a formal positive charge). *Journal of Physics and Chemistry of Solids* 46, 763–789 (1985).

21. Takeda, Y., Imayoshi, K., Imanishi, N., Yamamoto, O. & Takano, M. Preparation and characterization of Sr2 –xLaxFeO4(0 ⩽x⩽ 1). *J. Mater. Chem.* 4, 19–22 (1994).

22. Dann, S. E., Weller, M. T., Currie, D. B., Thomas, M. F. & Al-Rawwas, A. D. Structure and magnetic properties of Sr2FeO4 and Sr3Fe2O7 studied by powder neutron diffraction and Mössbauer spectroscopy. *J. Mater. Chem.* 3, 1231–1237 (1993).

23. Hinatsu, Y., Tezuka, K., Inamura, M. & Masaki, N. M. Magnetic Susceptibilities and Mössbauer Spectra of $Sr_{2-x}La_xFeO_{4-\delta}$ ($0\leq x\leq 0.5$). *Journal of Solid State Chemistry* 146, 253–257 (1999).
24. Burriel, M. *et al.* Absence of Ni on the outer surface of Sr doped La_2NiO_4 single crystals. *Energy Environ. Sci.* 7, 311–316 (2013).
25. Zhu, Y. *et al.* $SrNb_{0.1}Co_{0.7}Fe_{0.2}O_{3-\delta}$ Perovskite as a Next-Generation Electrocatalyst for Oxygen Evolution in Alkaline Solution. *Angew. Chem.* 127, 3969–3973 (2015).
26. Lee, J. G. *et al.* A New Family of Perovskite Catalysts for Oxygen-Evolution Reaction in Alkaline Media: $BaNiO_3$ and $BaNi_{0.83}O_{2.5}$. *J. Am. Chem. Soc.* 138, 3541–3547 (2016).
27. Grimaud, A. *et al.* Double perovskites as a family of highly active catalysts for oxygen evolution in alkaline solution. *Nat. Commun.* 4, ncomms3439 (2013).
28. Yamada, I., Murakami, M., Hayashi, N. & Mori, S. Inverse Charge Transfer in the Quadruple Perovskite $CaCu_3Fe_4O_{12}$. *Inorg. Chem.* 55, 1715–1719 (2016).
29. Ebrahimizadeh Abrishami, M. *et al.* Oxygen Evolution at Manganite Perovskite Ruddlesden-Popper Type Particles: Trends of Activity on Structure, Valence and Covalence. *Materials* 9, 921 (2016).
30. Jung, K.-N. *et al.* Doped Lanthanum Nickelates with a Layered Perovskite Structure as Bifunctional Cathode Catalysts for Rechargeable Metal–Air Batteries. *ACS Appl. Mater. Interfaces* 5, 9902–9907 (2013).
31. Jung, J.-I. *et al.* Optimizing nanoparticle perovskite for bifunctional oxygen electrocatalysis. *Energy Environ. Sci.* 9, 176–183 (2016).
32. Louie, M. W. & Bell, A. T. An Investigation of Thin-Film Ni–Fe Oxide Catalysts for the Electrochemical Evolution of Oxygen. *J. Am. Chem. Soc.* 135, 12329–12337 (2013).
